# Integrated Estimation of Stress and Damage in Concrete Structure Using 2D Convolutional Neural Network Model Learned Impedance Responses of Capsule-like Smart Aggregate Sensor

**DOI:** 10.3390/s24206652

**Published:** 2024-10-15

**Authors:** Quoc-Bao Ta, Ngoc-Lan Pham, Jeong-Tae Kim

**Affiliations:** Department of Ocean Engineering, Pukyong National University, 45 Yongso-ro, Nam-gu, Busan 48513, Republic of Korea; tabao838@pukyong.ac.kr (Q.-B.T.); pnlan@pukyong.ac.kr (N.-L.P.)

**Keywords:** electromechanical impedance method, PZT sensor, smart aggregate, two-dimensional convolutional neural network, concrete structure, stress and damage monitoring

## Abstract

Stress and damage estimation is essential to ensure the safety and performance of concrete structures. The capsule-like smart aggregate (CSA) technique has demonstrated its potential for detecting early-stage internal damage. In this study, a 2 dimensional convolutional neural network (2D CNN) model that learned the EMI responses of a CSA sensor to integrally estimate stress and damage in concrete structures is proposed. Firstly, the overall scheme of this study is described. The CSA-based EMI damage technique method is theoretically presented by describing the behaviors of a CSA sensor embedded in a concrete structure under compressive loadings. The 2D CNN model is designed to learn and extract damage-sensitive features from a CSA’s EMI responses to estimate stress and identify damage levels in a concrete structure. Secondly, a compression experiment on a CSA-embedded concrete cylinder is carried out, and the stress–damage EMI responses of a cylinder are recorded under different applied stress levels. Finally, the feasibility of the developed model is further investigated under the effect of noises and untrained data cases. The obtained results indicate that the developed 2D CNN model can simultaneously estimate stress and damage status in the concrete structure.

## 1. Introduction

Concrete structures are essential in construction because of their versatility and cost-efficiency. Over time, critical parts of concrete structures undergo degradation and damage under constant pressure. Thus, it is important to regularly monitor stress and damage status as a pre-requisite procedure at critical locations [1,2].

To date, several well-known non-destructive testing methods, such as X-ray scans [3], fiber-optic sensors [4], piezoelectric sensors [5], strain gauge sensors [6], and cement-based sensors [7], have been employed for keeping an eye on the health of concrete structures. Among these, strain-based methods [6], attached to concrete surfaces, are frequently utilized to monitor concrete stress and damage status. However, the sensitivity of surface-attached strain sensors in inner damage detection was limited due to small sensing regions [8].

The EMI technique has recently become popular among researchers due to several key benefits [9]. Firstly, the EMI technique can be applied to various structures such as civil infrastructures, mechanical systems, and aerospace structures. Secondly, the EMI technique is susceptible to minor structural damage because it uses the EMI response in a high-frequency range. Finally, the EMI technique is cost-effective and can be integrated with a real-time SHM measurement system. The EMI technique utilizes PZT transducers to interact with structures, providing insights into local characteristics [10]. Surface-mounted PZT sensors can detect surface damage but are less sensitive to inner concrete damage [11].

To reduce the effect of noisy ambient conditions and temperature variation on PZT sensors, Song et al. [12] developed an EMI-based cylindrical smart aggregate (SA) technique to detect concrete damage early. In 2017, Kong et al. [13] developed an EMI-based spherical smart aggregate (SSA) technique providing omnidirectional actuating and sensing capabilities. SSA sensors have proven to be superior to the earlier cylindrical smart aggregate sensors, which could only generate or receive stress waves in a single direction [13]. In general, SA sensors have been applied to monitor concrete structures for strength development [14], compressive stress and internal damage [15], and crack repair [16]. However, SA techniques need to find the sensitivity of the frequency band manually; in addition, the information provided by resonant frequency shifts is often unclear, and it is difficult to observe a trend in their results. To deal with these issues, Pham et al. [17] created a new and improved SA technique named the capsule-like smart aggregate (CSA). CSA sensors can monitor concrete health with a pre-determined frequency range of less than 100 kHz. In general, concrete stress changes and global and local damage occurrence can be reflected directly via EMI responses from SA sensors.

Despite their advantages, the main issues of the damage monitoring technique via SA sensors include efficiently managing data collection, effectively processing the gathered information, and accurately assessing structural damage. In the EMI technique, standard EMI features like root-mean-square deviation (RMSD) and cross-correlation deviation (CCD) are frequently utilized to monitor structure damage. Nonetheless, these traditional multi-step methods require a rigorous and effective feature extraction procedure to achieve precise quantitative assessments of stress and damage [18]. Selecting EMI features with bias and insufficient analysis can result in incorrect damage alerts. Nguyen et al. [19] showed the challenges of damage quantification using statistical indices because of the non-linear properties within EMI responses. When assessing damage in prestressed concrete structures, the statistical indices calculated in the subrange of the raw EMI response exhibited noticeable variations in certain damage cases, while in other cases, unexpected fluctuations were observed [20].

So far, recent studies have tried combining CNN-based regression algorithms with EMI techniques for the stress estimation of civil infrastructures. The effectiveness of the 1D CNN algorithm in independently learning damage-sensitive EMI features was evaluated to monitor damage in a prestressed reinforced concrete girder [21]. Lee et al. [22] proposed an autonomous crack detection algorithm using a 2D CNN to reliably detect cracks due to noise and undesired artifacts on damaged surfaces in the real world. Ta et al. [23] developed a 1D CNN deep regression learning model to process raw EMI responses measured by the SA sensor for monitoring concrete stress. In another study, Ta et al. [24] integrated the 1D CNN regression model with the CSA-based EMI measurement technique for monitoring concrete stress. The stress in the investigated concrete structure could be automatically estimated with high accuracy, even with noise effects and missing data.

Until now, CNN-based classification algorithms have been developed to estimate damage to civil infrastructures. For example, Nguyen et al. [20] proposed a 1D CNN deep learning approach using raw EMI data to monitor and assess bolt loosening in steel structures. In a different study, Nguyen et al. [25] applied the 1D CNN model to determine the PZT transducer’s damage status. Yan et al. [26] introduced a feature extraction network using an EMI-integrated 1D CNN model to assess the early-age hydration of cement mortar. Their method was better at quantifying changes in EMI response compared to older machine learning methods. Ai et al. [27] introduced a basic 2D CNN method for damage detection in a cubic concrete structure. In a subsequent study, Ai and Cheng [28] segmented the EMI signatures into subrange responses. The ranges were processed by a statistical approach to fit the 2D CNN’s input for concrete damage quantification. Their experiments indicated that the proposed CNN models attained high accuracy, even when detecting small damages. Recently, Ai et al. [29] proposed a 1D CNN approach for processing raw admittance responses to automatically detect small-size damages in concrete structures. As compared to a traditional backpropagation neural network, their proposed 1D CNN model demonstrated a significantly higher prediction accuracy.

Despite these research efforts, there are two remaining issues. Firstly, a deep learning-based damage measurement method for processing EMI signals of an embedded CSA sensor has not been developed so far. Secondly, the existing studies have separately focused on stress estimation by CNN regression or damage estimation by CNN classification. The mechanism behaviors of a CSA-embedded concrete structure under compression have not been fully investigated. To address these issues, this paper proposed a 2D CNN regression and classification model that learned the CSA-based stress–damage EMI responses to estimate concrete stress and damage.

The major contributions of this work lie in the following: (1) the 2D CNN-based deep learning of the CSA’s stress–damage EMI responses for concrete damage monitoring is firstly studied; (2) a 2D CNN-based deep regression and classification model is developed for stress estimation and damage identification in concrete structures; and (3) a performance evaluation of the 2D CNN model under the effects of noise-contaminated and untrained stress–damage EMI data on concrete damage monitoring is conducted.

This study proposes a 2D CNN model that learned the EMI responses of the CSA sensor to integrally estimate stress and damage in concrete structures. Firstly, the overall scheme of this study is described. The CSA-based EMI technique is theoretically presented by describing the behaviors of a CSA sensor embedded in a concrete body under compressive loadings. A 2D CNN model is designed to learn and extract damage features from the CSA’s EMI signals for estimating stress and identifying damage levels in a concrete structure. Secondly, a compression experiment on a CSA-embedded concrete cylinder is carried out, and the stress–damage EMI signals of the cylinder are recorded under different applied stress levels. Finally, the feasibility of the developed model is further investigated under the effect of noises and untrained data cases.

## 2. Methodology

### 2.1. Scheme of Concrete Damage Monitoring

**Figure 1:**
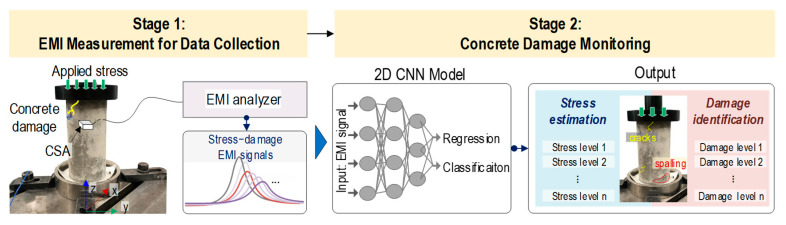
Scheme of CSA-based concrete damage monitoring by 2D CNN model.

Figure 1 presents an integrated scheme of CSA-based concrete damage monitoring via the 2D CNN deep learning of EMI responses. The scheme has two stages: (1) EMI measurement for data collection and (2) concrete damage monitoring.

In Stage 1, a concrete cylinder is subjected to a series of applied compression stresses. During the compression test, the EMI responses and corresponding structural attributes (i.e., stress and severity of concrete damage) are recorded to create datasets for the 2D CNN model. The characteristics of each attribute lead to different deep learning approaches. The stress levels exhibiting continuous behavior are suitable for 2D CNN deep regression learning. The severity of damage expressed in the discrete behavior for the damage phase is suitable for 2D CNN deep classification learning. Note that the observed concrete damage in concrete is due to local damage propagation, which occurs when the applied stress reaches the yield condition of the concrete material [30].

In Stage 2, a 2D CNN deep learning model integrated with regression and classification layers is developed for automatically processing stress–damage EMI signals. With each input of the stress–damage EMI response into the model, the model simultaneously outputs the concrete stress estimation through the regression layer and concrete damage identification through the classification layer. This integrated approach leverages the powerful feature extraction capabilities of the 2D CNN model to decode complex patterns induced by the damage existent in EMI signals, providing a comprehensive assessment and maintenance for concrete structures.

### 2.2. CSA-Based EMI Measurement Technique

**Figure 2:**
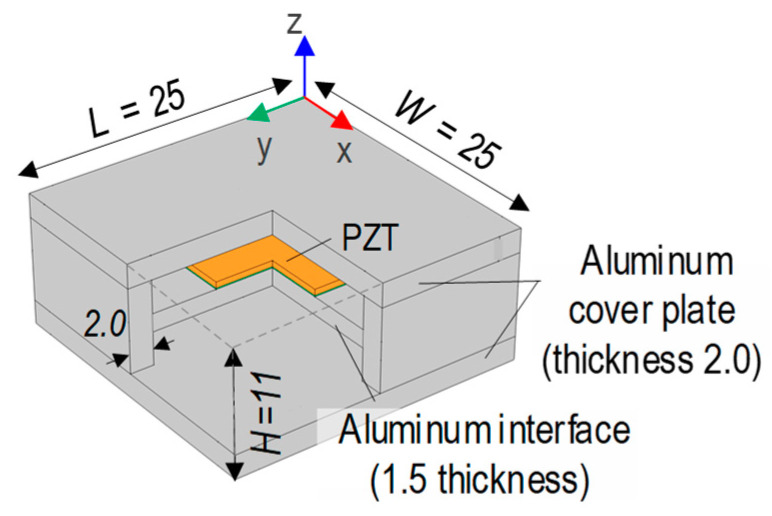
Prototype of capsule-like smart aggregate (CSA).

Figure 2 shows the CSA-based EMI measurement technique for concrete damage monitoring [17]. The CSA sensor prototype has dimensions of 25 mm in length (L), 25 mm in width (W), and 11 mm in height (H). The CSA sensor is composed of a PZT patch, an aluminum interface, and two aluminum cover plates.

The selected PZT material is PZT-5A due to its sensitivity in damage detection [31]. The aluminum interface plate, the so-called vibrating plate, is a flexible aluminum plate with fixed ends to the CSA’s wall (thickness 2 mm). The vibrating plate identifies the sensitive frequency band of the PZT sensor [32]. The PZT sensor is attached to the interface via an epoxy glue layer (i.e., bonding layer) with a size of 10 × 10 × 0.1 mm. The aluminum cover plates are adhered to the walls to shield the PZT sensor during the CSA-embedded concrete fabrication process.

**Figure 3:**
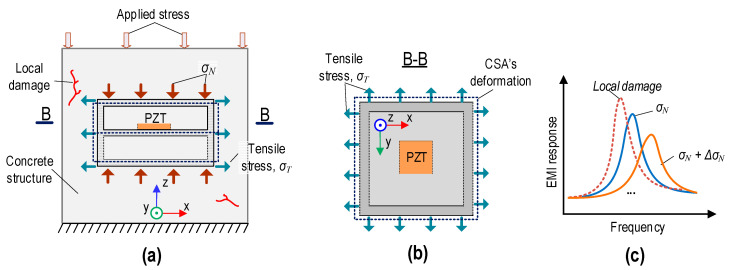
Behaviors of EMI responses of CSA embedded in concrete structure under applied stress in z-direction: (**a**) CSA in structure under compression; (**b**) Section B-B; (**c**) changes in EMI responses.

Figure 3 presents the behavior of the EMI responses of a CSA embedded in a concrete structure under compression. Under applied stress in the z-direction, the CSA sensor embedded in the concrete structure experiences stress–strain responses (see Figure 3a). Due to the applied stress, the CSA’s surfaces are subjected to compressive stress (σ*_N_*) along the vertical direction (i.e., z-direction). Due to Poisson’s effect, the other CSA’s surfaces (i.e., y-direction and x-direction) are subjected to tension stress (σ*_T_*).

When the compressive stress acting on the CSA is increased along the z-direction, this makes the vibrating plate (see Figure 3b) expand in the y-direction and x-direction. The expansion of the vibrating plate leads to changes in the structural impedance. Therefore, increasing the applied stress in the CSA-embedded concrete structure could lead to a rightward shift in EMI responses (see Figure 3c). 

For increasing stress (e.g., σ*_N_* + Δσ*_N_*), local damage may occur because stress reaches the yield condition threshold of the concrete material. When this threshold is exceeded, micro-cracks or other forms of damage initiate within concrete, particularly around the critical stress area near the CSA’s position. Damage makes concrete’s mechanical properties (e.g., natural frequencies, stiffness, and damping ratios) change. In addition, damage causes the tensile stress acting on the vibrating plate to release rapidly [17]. This phenomenon indicates that the concrete medium around the CSA undergoes a transformation, leading to abrupt changes in the EMI responses [33], as shown in Figure 3c.

### 2.3. Databank of Stress–Damage EMI Signals

EMI signals may undergo changes due to diverse factors like sensor configuration and sensor bonding [34]. Conducting comprehensive experiments accounting for these factors poses expenses and challenges. Hence, data augmentation like adding Gaussian noise to EMI signals is an effective approach to simulate real-world measurement conditions. In addition, deep learning techniques regularly face challenges in maintaining stability and accuracy [35] when they learn small data. A well-constructed deep learning model maintains high performance despite using small datasets.

**Figure 4:**
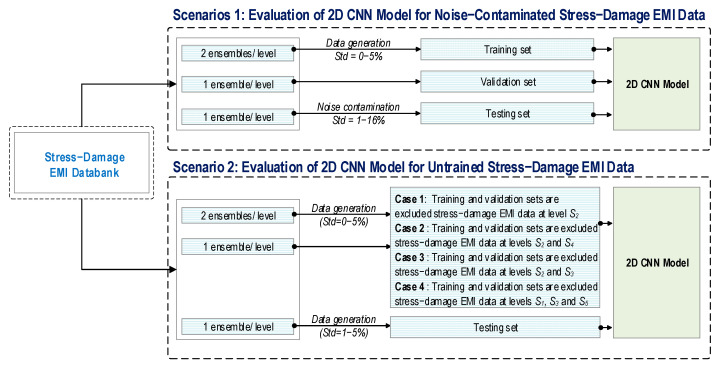
Data configuration of stress–damage EMI data for 2D CNN model.

As shown in Figure 4, two scenarios are established to deal with the above-mentioned issues by evaluating the performance of a stress estimation and damage identification integrated 2D CNN model. In Scenario 1, the effect of noise-contaminated stress–damage EMI data on the robustness of the proposed 2D CNN model is evaluated. To construct the training set, Gaussian noise with standard deviations of 0%, 1%, 2%, 3%, 4%, and 5% of the stress–damage EMI amplitude is added to the first two ensembles (among four ensembles) in each applied stress level. The third ensemble tracks the overfitting status [36] during training. To construct the testing set, the last ensemble at each stress level is added with different noise levels ranging from 1% to 16% in 1% increments.

In Scenario 2, the effect of the untrained stress–damage EMI data on the robustness and generalization of the proposed 2D CNN model is evaluated. Similar to Scenario 1, the first two ensembles have added noises with a standard deviation from 0% to 5% of signal magnitude to generate a training dataset for the 2D CNN model. The third ensemble is utilized to validate the model. The last ensemble is added noise with a standard deviation from 0% to 5% of signal magnitude to construct a testing set. 

To investigate the effect of untrained stress and damage levels, two cases are established for evaluating the performance of the 2D CNN model. In Case 1, the training and validating sets 1 are established by excluding the stress–damage EMI dataset at stress level S_2_ (among five levels S_1−5_). In Case 2, the training and validating sets 2 are established by excluding the stress–damage EMI data at levels S_2_ and S_4_. In Case 3, the training and validating sets 3 are established by excluding the stress–damage EMI data at levels S_2_ and S_3_. In Case 4, the training and validating sets 4 are established by excluding the stress–damage EMI data at levels S_1_, S_3_, and S_5_. 

### 2.4. Design of 2D CNN Deep Regression and Classification Model

#### 2.4.1. Architecture of 2D CNN Model

**Figure 5:**
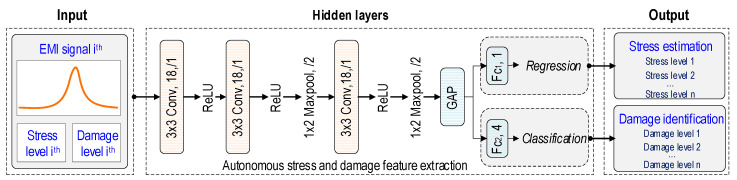
Architecture of 2D CNN deep regression and classification model.

Figure 5 depicts the architecture of a deep learning model using the CSA’s raw EMI signals. A 2D CNN-based deep regression and classification learning model is designed for stress estimation and damage identification in concrete structures. The architecture design of the model is inspired by a previous study [37], and the hyper-parameters are fine-tuned following the practical guidance by Zhang and Wallace [38]. A preliminary study was conducted to select an appropriate architecture for the 2D CNN model, as presented in Appendix A. Three 2D CNN architectures (M1–M3) with different depths are designed, and their training performances are compared. According to the comparison results, the best architecture (M3) is selected for concrete stress estimation and damage identification, as depicted in Figure 5.

The architecture of the 2D CNN model consists of an input layer, a series of hidden layers, and an output layer. The input layer accepts EMI data in the format of N × 15 × 15, where N is the number of raw EMI signals, and array 15 × 15 is transformed from 225 measurement points of each EMI signal. Each EMI signal is labeled with a corresponding status of the concrete structure (i.e., stress and damage level). The stress levels exhibiting continuous behavior are used for regression learning. The damage levels exhibiting discrete behavior are used for classification learning. Details on the stress–damage EMI signals are found in Section 3.3.

Secondly, the hidden layer includes three Conv layers, three ReLU layers, two Maxpool layers, one global average pooling (GAP) layer [39], two fully connected (Fc) layers, a regression output (Regression) layer, and a classification output (Classification) layer. The Conv layers with a kernel size of 3 × 3 apply convolution operations to the EMI input data to extract stress–damage EMI features. The ReLU layers apply a non-linear activation function that replaces all negative values in the feature maps (e.g., Conv layer’s output) with zero, allowing the model to learn non-linear relationships. The Maxpool layers with a kernel size of 2 × 2 downsample the feature maps by selecting the maximum value from small patches. The proposed 2D CNN model has 6143 training parameters. Information on the layers of the 2D CNN model is outlined in Table 1. The GAP layer following the Maxpool layer is used to average the feature maps. 

The Fc layers merge and reshape the extracted features into low-dimensional features suitable for the regression and classification layers. There is 1 neuron in the layer Fc_1_, and it connects directly to the regression layer. Fc_2_ has 4 neurons, and these neurons connect directly to the classification layer. Finally, the output includes “Stress estimation” and “Damage identification”. As shown in Figure 5, the regression layer is in charge of predicting the concrete stress levels, while the classification layer is responsible for identifying the levels of concrete damage status.

#### 2.4.2. Evaluation of Deep Regression Learning

As given in Equation (1), the loss function for the regression layer, *L_reg_*, is used to calculate the prediction error in terms of stress. Here, *n* is the total number of data. The parameters $\sigma_{i}$ and ${\hat{\sigma }}_{i}$ represent the predicted and actual stress values for the *i*^th^ data point. The loss regression value (*L_reg_*) indicates the error in stress monitoring with the MPa unit.

(1) $$L_{reg}=\frac{1}{n}\overset{n}{\underset{i=1}{\sum }}\left|\sigma_{i}-{\hat{\sigma }}_{i}\right|$$

As given in Equation (2), the regression accuracy of the trained 2D CNN model was evaluated by using the root-mean-square error (RMSE) index. The RMSE calculates the average squared difference between predicted stress $\sigma_{i}$ and actual stress ${\hat{\sigma }}_{i}$. The RMSE with a unit of MPa represents the accuracy of stress prediction.

(2) $$\mathrm{RMSE}=\sqrt{\frac{1}{N}\overset{N}{\underset{i=1}{\sum }}{\left(\sigma_{i}-{\hat{\sigma }}_{i}\right)}^{2}}$$

#### 2.4.3. Evaluation of Deep Classification Learning

As given in Equation (3), the loss function of the classification layer, *L_class_*, represents the categorical cross-entropy loss [40]. The symbol m denotes the number of labeled damage levels. $p_{i}^{j}$ and ${\hat{p}}_{i}^{j}$ are the predicted probability and true probability for the *j*^th^ damage level. These probability values are calculated via a softmax activation function [40]. Briefly, the loss function of the classification layer measures the dissimilarity between the predicted distribution $p_{i}^{j}$ and the true probability distribution ${\hat{p}}_{i}^{j}$.

(3) $$L_{class}=\frac{1}{n}\overset{n}{\underset{i=1}{\sum }}\left(\overset{m}{\underset{j=1}{\sum }}{\hat{p}}_{i}^{j}\log\left(p_{i}^{j}\right)\right)$$

A confusion matrix chart is utilized to evaluate the classification accuracy of the trained 2D CNN model on the testing dataset. The diagonal cells indicate correct classification, denoted as true positives (TPs). The off-diagonal cells represent incorrect prediction, denoted as false negatives (FNs) and false positives (FPs). As described in Equation (4), the true positive rate (TPR), false negative rate (FNR), precision, false discovery rate (FDR), and accuracy are defined as follows:

TPR = TP/(TP + FN) FNR = FN/(TP + FN) Precision = TP/(TP + FP) FPR = FP/(TP + FP) Accuracy = TP/(TP + FP + FN)(4)

For the proposed 2D CNN model dealing with regression and classification tasks simultaneously, a representative loss is calculated as *L_total_* = *L_reg_* + *L_class_*, where *L_reg_* and *L_class_* are the loss functions of the regression and classification layers. The accuracy of the trained 2D CNN is evaluated by regression metrics and classification metrics. 

## 3. Experimental Test

### 3.1. Fabrication of CSA-Embedded Concrete Cylinder

**Figure 6:**
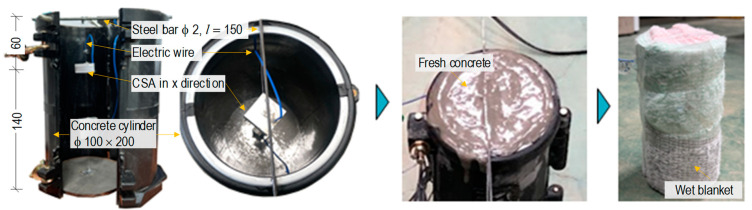
Fabrication of CSA-embedded concrete cylinder.

Figure 6 shows the fabrication process of a CSA-embedded concrete cylinder. The CSAs were positioned at two locations of a cylinder mold with a size of 100 × 200 mm. The CSA with a normal PZT vector along the z-direction was called a z-CSA sensor. 

The z-CSA was spaced at a distance of 140 mm from the bottom of the cylinder mold. To position the CSA sensor in the cylinder mold, plastic wires and a steel bar (2 mm in diameter, 150 mm in length) were used. After weighing and mixing the concrete ingredients, they were poured into the mold. The mold was removed 48 h after pouring, and the concrete sample was cured for 28 days under normal conditions using a wet blanket. The concrete sample was tested at 28 days of age. The material properties of the concrete cylinder and CSA sensor are detailed in Table 2.

### 3.2. Experimental Setup

**Figure 7:**
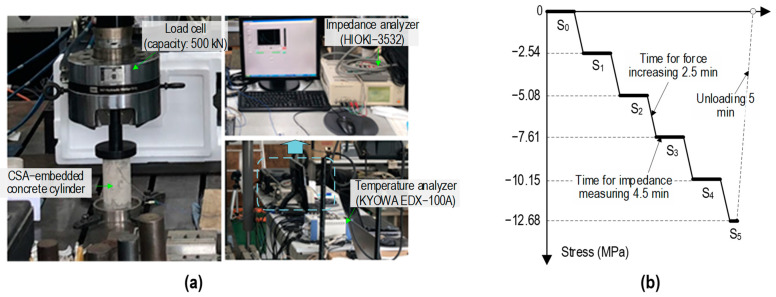
Experimental setup: (**a**) stress–damage EMI measurement in CSA-embedded cylinder under compressive load; (**b**) compressive loading scenario.

Figure 7 presents the setup of a concrete cylinder under compression forces. The concrete cylinder was inserted into the load frame of the MTS system (servo-hydraulic materials testing version 793). A load cell (capacity up to 500 kN) was used to monitor the real compression force. An impedance analyzer, HIOKI 3532-50 LCR HiTESTER, was used to measure stress–damage EMI signals from the CSA sensor. A KYOWA EDX-100A was used to measure the room temperature.

Prominent peaks in the EMI responses represented meaningful structural information [17]. It was noted that the CSA sensor in the cylinder showed a prominent peak in EMI responses from 15 kHz to 26 kHz. The EMI responses were swept between 15 kHz and 26 kHz with 224 intervals to determine the sensitivity of the embedded CSA’s EMI responses to compression loading. The measured EMI responses and corresponding structural attributes (i.e., stress levels and concrete damage levels) were recorded to build a stress–damage EMI dataset for the evaluation of the 2D CNN deep regression and classification learning model. The measured temperature fluctuated between 22 °C and 23 °C (with a difference of approximately 1 °C). Consequently, the temperature effect on the EMI responses was disregarded. 

As shown in Figure 7b, six loading scenarios (S_0_ = 0 MPa to S_5_ = 12.68 MPa in steps of 2.54 MPa) were implemented in the CSA-embedded cylinder. Stress was applied in constant increments, with 2.5 min for stress increases and 4.5 min for EMI monitoring. The loading rate was consistently maintained at 0.0113 MPa·s^−1^.

### 3.3. Stress–Damage EMI Signatures of CSA-Embedded Concrete Cylinder

**Figure 8:**
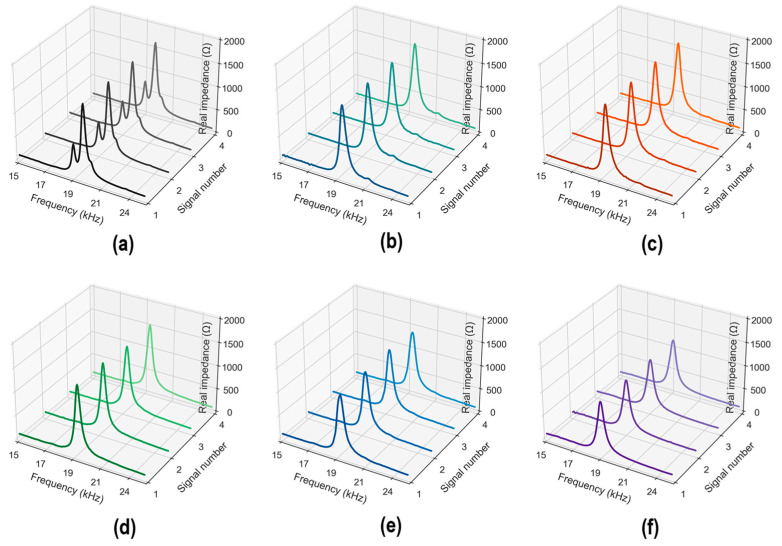
Measured stress–damage EMI responses of z-CSA under applied stresses: (**a**) S_0_; (**b**) S_1_; (**c**) S_2_; (**d**) S_3_; (**e**) S_4_; (**f**) S_5_.

Figure 8 shows the measured stress–damage EMI responses of the z-CSA sensor under applied stress levels S_0–5_. In each stress level, four EMI measurements (four ensembles) were repeatedly recorded in a frequency range of 15–26 kHz. As shown in Figure 8a, four EMI ensembles under applied stress S_0_ were plotted. The peak of ensembles was captured clearly under a frequency range of 15–26 kHz. For increasing stress levels, the stress–damage EMI’s resonant peak frequency and real magnitude varied significantly (see Figure 8b–f).

**Figure 9:**
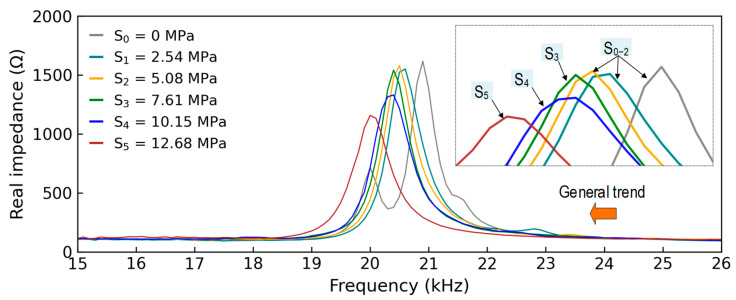
Stress–damage EMI responses of z-CSA under applied stresses S_0–5_.

Figure 9 shows the average stress–damage EMI responses in four ensembles to investigate variations in the peak frequency and peak magnitude. There was a reduced trend in the frequency and magnitude of the resonant peak under increasing applied stress. The resonant peak of the CSA sensor shifted leftward. The variation trend in the experimental stress–damage EMI signals was inconsistent with the theoretical explanation in Section 2.2. This could be caused by the high compressive stress applied to the CSA sensors during the concrete strength development, the non-homogeneous feature of concrete materials, and the CSA fabrication method. 

**Figure 10:**
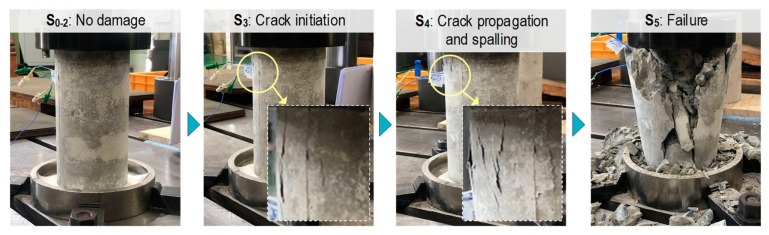
Visual observation of test specimen under applied stresses S_0–5_.

The damage levels in the test specimen under applied stresses S_0–5_ are shown in Figure 10. Crack imitation was found at loading level S_3_. Crack propagation and concrete spalling were observed at loading level S_4_. Then, the concrete damage developed and failed at loading level S_5_.

### 3.4. Statistical Quantification of Stress–Damage EMI Responses

The stress–damage EMI responses of the z-CSA-embedded concrete cylinder under compressive tests were utilized to calculate stress–damage EMI features (RMSD and CCD [41]). The first stress level S_0_ (at 0 MPa) was neglected in EMI feature computation due to the uncertainty effect in experimental measurements. The upper control limit (UCL) threshold was established to aid decision-making. The UCL threshold was computed from three standard deviations of the mean, corresponding to a 99% confidence level. Any EMI features above the UCL indicate changes in the applied stress level. 

**Figure 11:**
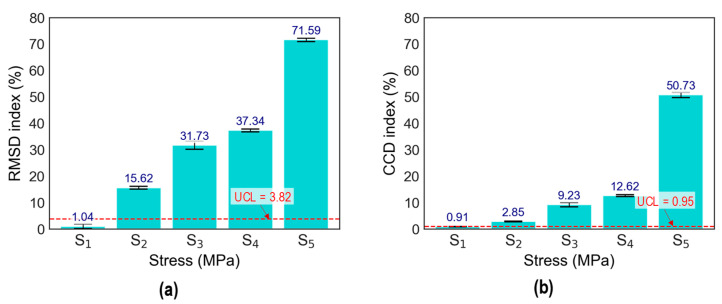
Stress–damage EMI features of CSA under applied stresses S_1–5_: (**a**) RMSD; (**b**) CCD.

Figure 11 shows the stress–damage EMI features of CSA under applied stresses (S_1_–S_5_). As shown in Figure 11a, the RMSD indices increased non-linearly as the stress levels increased. Figure 11b shows a non-linear increase in CCD indices with ascending stress levels from S_2_ to S_5_. Note that the RMSE and CCD indices of the CSA displayed sudden variations at stress level S_3_. This indicated that an alteration from local damage to external cracks of the concrete cylinder was found [33]. 

The analysis of the statistical metrics revealed that the RMSD and CCD indices exhibited greater sensitivity to EMI’s variations under applied stress levels. Although the mentioned statistical indies could quantify damage to the concrete structure, they required multiple steps of data processing and were not automated for stress estimation and damage identification. Therefore, there is a need to develop an automated stress and damage estimation technique. 

## 4. Evaluation of 2D CNN-Based Deep Regression and Classification Model

### 4.1. Stress and Damage Monitoring for Noise-Contaminated Stress–Damage EMI Data

#### 4.1.1. Data Preparation

The measured stress–damage EMI data of the concrete cylinder embedded CSA sensor was utilized to configure the databank for the 2D CNN deep regression and classification learning model. The first stress level S_0_ (at 0 MPa) was neglected for the 2D CNN model due to the uncertainty effect in experimental measurements. 

Table 3 shows the labels of “stress” and “damage level” assigned to the measured stress–damage EMI data. For the stress regression task, the five concrete stress levels from S_1_ to S_5_ were labeled with stress values from 2.53 MPa to 12.68 MPa with an interval of 2.53 MPa. For the damage identification task, four concrete damage levels, including “No damage”, “Crack initiation”, “Crack propagation and spalling”, and “Failure” corresponding to labels “DL0”, “DL1”, “DL2”, and “DL3”, were assigned to the measured stress–damage EMI data. 

The influence of noise levels on the proposed 2D CNN model’s performance was monitored through the training, validation, and testing datasets generated from the stress–damage EMI data (see Figure 4). For the compressive test on the cylinder, the EMI signal was repeatedly measured four times (four ensembles) in each applied stress level. In total, 20 signals were acquired from 5 applied stress levels. To construct the training set, the first two ensembles (among four ensembles) in each applied stress level were added Gaussian noise levels with standard deviations of 0%, 1%, 2%, 3%, 4%, and 5% of the stress–damage EMI amplitude. For each stress level, stress–damage EMI signals were generated four times. As a result, there were a total of 240 signals (2 signals × 5 stress levels × 6 noise levels × 4 times) for 5 applied stress levels. The third ensemble was utilized to construct the validation set, and there were five total signals in the validation set. To create the testing dataset, the last ensemble in each applied stress level was added noise levels from 1% to 16% with 1% increments. For each stress level, 10 new signals were generated. As a result, we generated a total of 800 EMI signals for the 16 noise levels. Combined with the 5 original EMI signals, this resulted in a testing dataset of 805 signals (5 signals × 16 noise levels × 10 times + 5 original signals).

**Figure 12:**
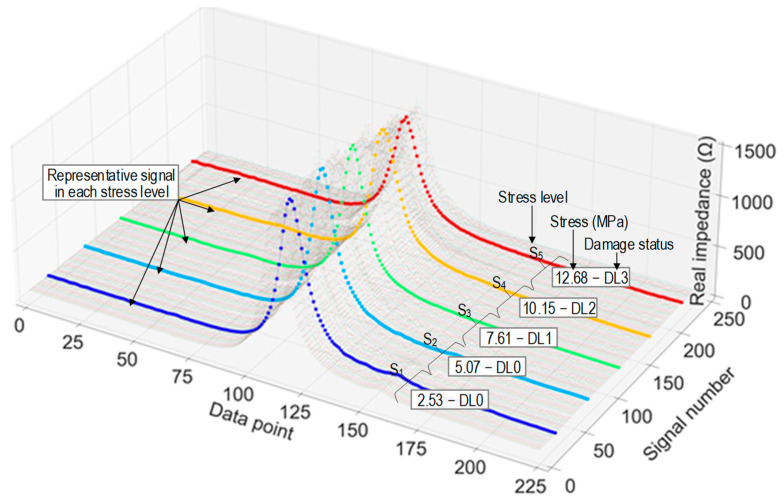
EMI responses (in average of ensembles) CSA in cylinder under applied stresses S_1–5_.

**Figure 13:**
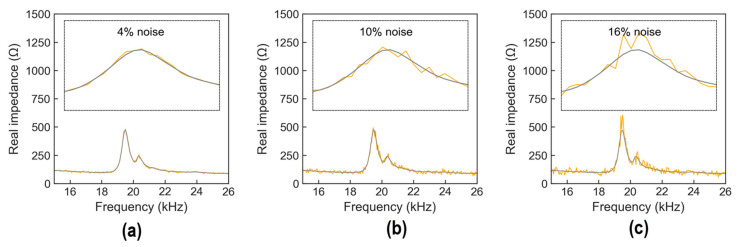
Example of noise-contaminated stress–damage EMI signals under applied stress level S_1_ in testing set: (**a**) 4% noise; (**b**) 10% noise; (**c**) 16% noise.

Figure 12 shows a visualization of the stress–damage EMI responses as labeled in the training set. In the figure, each EMI signal was plotted with 225 data points. Corresponding to each stress level, there were 10,800 data points. As a result, 54,000 data points were obtained corresponding to 5 stress levels. Figure 13 shows an example of noise-contaminated stress–damage EMI signals under stress level S_1_ in a testing set. 

#### 4.1.2. Training Results

The computations were conducted on a desktop computer (RAM—64 GB, CPU—Intel Core i9-9000KF of 3.6 GHz, GPU—GeForce GT 2080 Ti of 11 GB). The 2D CNN model was constructed using Python programming language version 3.9. The Adam optimizer with a learning rate of 0.0001 and a mini-batch size of 2 were utilized to train the model. 

**Figure 14:**
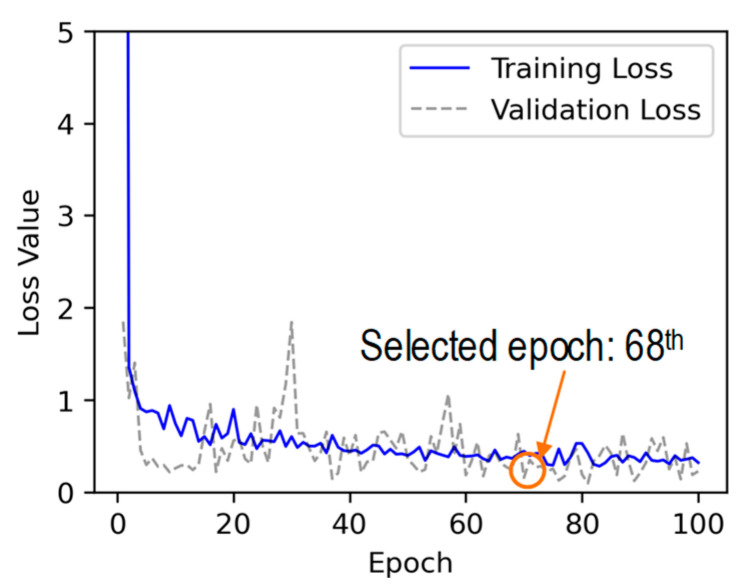
Loss values of 2D CNN model after 100 epochs.

The training and validation loss values of the 2D CNN model during a training procedure of 100 epochs are plotted in Figure 14. The loss values quickly decreased within the initial 6 epochs. After that, the loss values continued to decrease until the 100^th^ epoch. It took 35.1 s to train the 2D CNN model with 6143 training parameters. It was observed that the lowest loss validation value in the whole learning process was at the 68^th^ epoch. Hence, the trained model at the 68^th^ epoch was selected to investigate its accuracy on the testing set. 

#### 4.1.3. Stress Estimation

**Figure 15:**
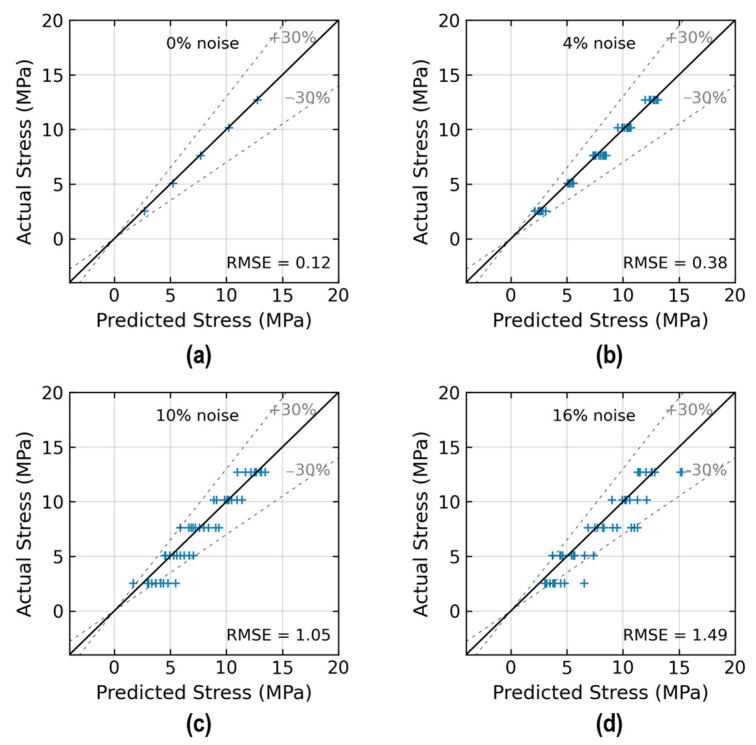
Stress prediction by 2D CNN model: (**a**) 0% noise; (**b**) 4% noise; (**c**) 10% noise; (**d**) 16% noise.

Figure 15 shows the accuracy of stress estimation by the trained 2D CNN model. The figure compares the predicted stress values with the actual values across different noise levels. With the 0% noise level (see Figure 15a), the model precisely estimated stress values with an RMSE of approximately 0.12. With the 10% noise level (see Figure 15c), the model precisely estimated stress values with an RMSE of 1.05. The prediction error of the 2D CNN was over 30% at the 10% noise level.

**Figure 16:**
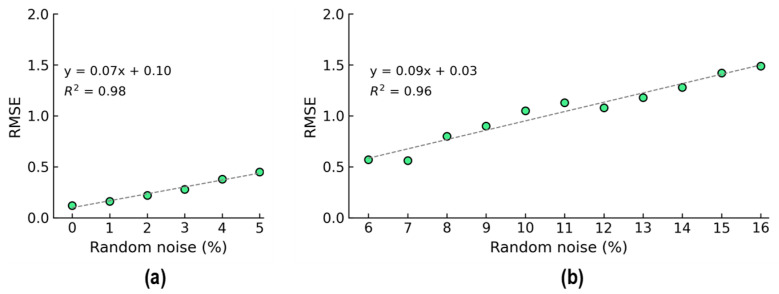
RMSE comparison of 2D CNN model for stress estimation. (**a**) Trained levels of noise (0–5%); (**b**) untrained levels of noise (6–16%).

The relationship between the RMSE and noise levels is plotted in Figure 16. As shown in Figure 16a, the RMSE was recorded as 0.1 at the 0% noise level, and it was recorded as 0.5 at the 5% noise level. As shown in Figure 16b, the RMSE was recorded as 0.57 at the 6% noise level, and it was recorded as 1.49 at the 5% noise level. It was observed that the increase in prediction error for untrained noise levels was significantly higher than that of trained noise levels, as indicated by the gradients of the linear approximation functions in Figure 16a,b. Generally, the accuracy of stress prediction diminished as the noise level increased in the 2D CNN model. 

As shown in Figure 16, the sum of the squared differences (R^2^) was close to one. Thus, the proposed model’s accuracy was less susceptible to the effects of increasing noise levels. The empirical function of the 2D CNN is encouraged for concrete stress prediction in practical measurement applications. 

#### 4.1.4. Damage Identification

**Figure 17:**
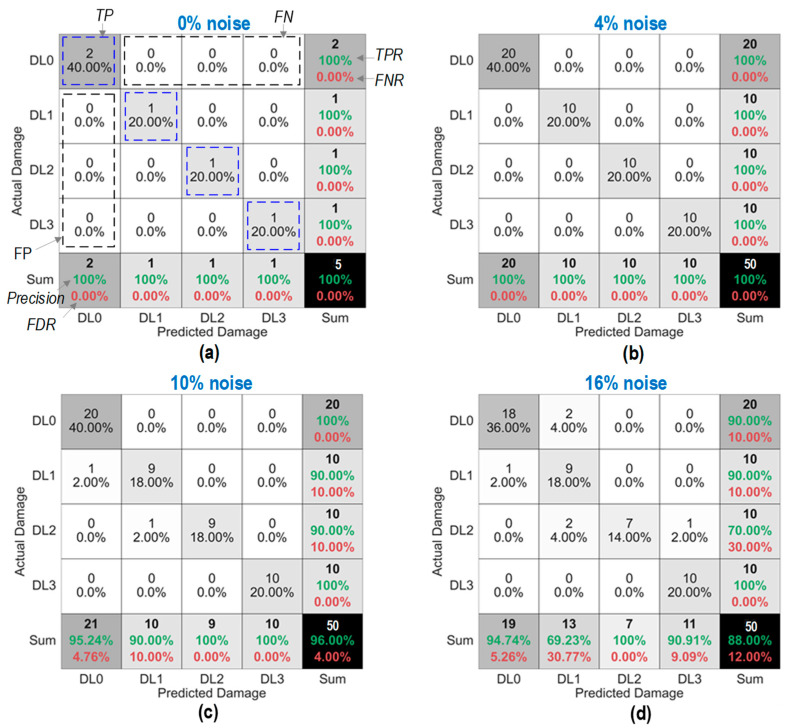
Damage identification of 2D CNN model under noise levels: (**a**) 0% noise; (**b**) 4% noise; (**c**) 10% noise; (**d**) 16% noise.

Figure 17 shows the accuracy of damage identification by the trained 2D CNN model. In each figure, the predicted damage was compared with the actual damage via a confusion matrix chart. With the 0% noise level (see Figure 17a), the model detected all damage statuses “DL0–DL3” exactly without false alarms. The overall accuracy of the model was 100%. With the 4% noise level (see Figure 17b), the model detected all damage levels “DL0–DL3” exactly. The overall accuracy of the model was 100%. 

With the 10% noise level (see Figure 17c), 100% of “DL0”, “DL1”, and “DL3” was accurately classified, and 90.0% of “DL2” was correctly classified. The precision for “DL1” reduced to 90.0%, and that of “DL2” and “DL3” remained unchanged at 100%. The overall accuracy of the model accounted for 96.0%. With the 16% noise level (see Figure 17d), the number of misclassifications was increased for “DL0” and “DL2”. The overall accuracy dropped to 88.0%, with 1–3 misclassifications for damage levels “DL0–DL2”.

It was observed that the precision of identified damage levels “DL0”, “DL1”, and “DL3” by the 2D CNN model was unstable. In brief, the overall accuracy of the 2D CNN model for damage classification was reduced when the noise level increased.

**Figure 18:**
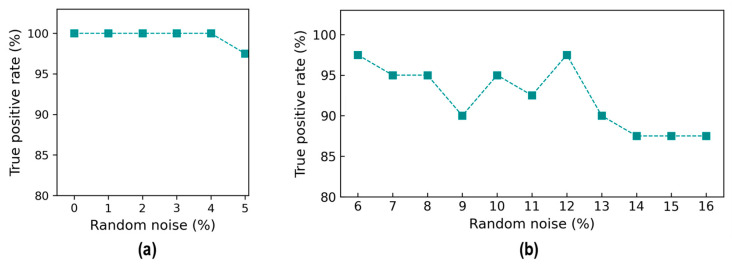
Accuracy of damage identification by 2D CNN model: true positive rate. (**a**) Trained levels of noise (0–5%); (**b**) untrained levels of noise (6–16%).

The accuracy metrics of damage identification were assessed for the effect of noise levels. Figure 18 shows the true positive rate (TPR) values of damage identification by the trained 2D CNN model. The TPR values reached 100% from the 0% to 4% noise levels, and it reduced to about 98.1% at 5% noise. The TPR values were 97.5% at the 6% noise level, followed by a variation around 95% until 12% noise. The TPR continuously reduced and reached 87.5% at 16% noise. It was observed that the reduction trend in the TPR was non-linear, and the changes were unstable. This could be induced by the effect of random noise injected in the testing set. 

**Figure 19:**
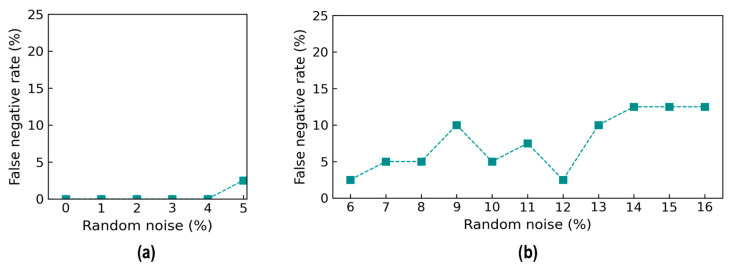
Accuracy of damage identification by 2D CNN model: false negative rate. (**a**) Trained levels of noise (0–5%); (**b**) untrained levels of noise (6–16%).

Figure 19 shows the false negative rate (FNR) values of damage identification by the trained 2D CNN model. The FNR was 0% from the 0% to 4% noise levels, and it started to increase to 2.0% at the 5% noise level. The FNR was 2.5% at the 6% noise level, followed by an increase of around 5% at the 10% noise level, and it gradually rose to 12.5% at the 14% noise level. After that, the FNR remained stable at 12.5% until 16% noise. The FNR fluctuated highly in noise levels ranging from 9 to 13% because of the incorrect identification for damage levels “DL1” and “DL2”.

**Figure 20:**
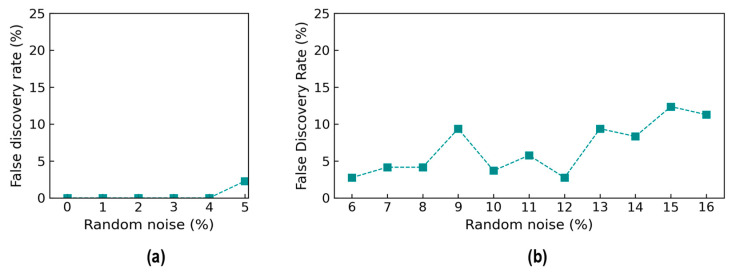
Accuracy of damage identification by 2D CNN model: false discovery rate. (**a**) Trained levels of noise (0–5%); (**b**) untrained levels of noise (6–16%).

Figure 20 shows the accuracy of damage identification by the trained 2D CNN model in terms of the false discovery rate (FDR). The FDR started to increase from 0% at the 0% noise level to 2% at the 5% noise level. The FDR accounted for 2.5% at the 6% noise level, followed by a slight fluctuation around 10% at the 12% noise level, and it rose to 12.5% at the 16% noise level.

### 4.2. Stress and Damage Monitoring for Untrained Stress–Damage EMI Data

#### 4.2.1. Data Preparation

Similar to the previous section, stress–damage EMI signals were repeatedly recorded with four ensembles for each applied stress level. A total of 20 signals were obtained from 5 stress levels. For each stress level, the first two ensembles (among four ensembles) were used to establish a total of 240 signals for 5 stress levels. The third ensemble was used for the validation set. This resulted in a total of five signals for five stress levels. The last ensemble was used to establish the testing set. This resulted in a total of 255 signals for 5 applied stress levels. 

**Figure 21:**
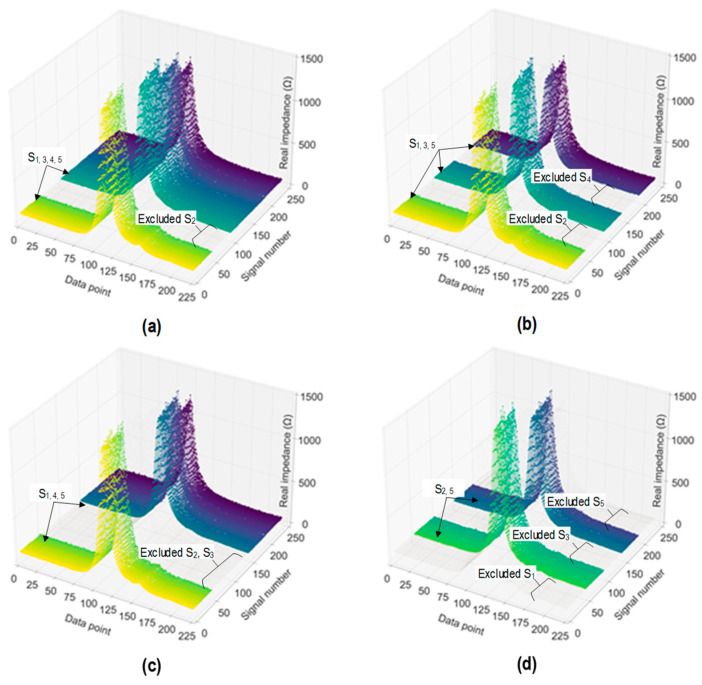
Visualization of labeled stress–damage EMI signals in training set for 2D CNN model: (**a**) Case 1 (untrained S_2_); (**b**) Case 2 (untrained S_2_, S_4_); (**c**) Case 3 (untrained S_2_, S_3_); (**d**) Case 4 (untrained S_1_, S_3_, S_5_).

As outlined in Table 4, four cases were established for untrained stress–damage EMI data. The effect of the untrained stress–damage EMI data was assessed on the performance of the 2D CNN model. Figure 21 visualizes the stress–damage EMI data labeled in the training set. In Case 1 (see Figure 21a), stress level S_2_ was excluded to establish the data for training and validating the trained 2D CNN model. A total of 48 signals at stress level S_2_ were excluded in the training set, and 1 signal was excluded in the validation set. As a result, training set 1 contained 192 signals, and validation set 1 included 4 signals. In Case 2 (see Figure 21b), stress levels S_2_ and S_4_ were excluded from the training set. By excluding stress levels S_2_ and S_4_, 96 signals and 2 signals were excluded in the training and validation sets, respectively. As a result, training set 2 contained 144 signals, and validation set 2 included 3 signals.

In Case 3 (see Figure 21c), stress levels S_2_ and S_3_ were excluded from the training set. By excluding stress levels S_2_ and S_3_, 96 signals and 2 signals were excluded in the training and validation sets, respectively. As a result, training set 3 contained 144 signals, and validation set 3 included 3 signals. In Case 4 (see Figure 21d), stress levels S_1_, S_3_, and S_5_ were excluded from the training set. By excluding stress levels S_1_, S_3_, and S_5_, 144 signals and 3 signals were excluded in the training and validation sets, respectively. As a result, training set 4 contained 96 signals, and validation set 4 included 2 signals.

#### 4.2.2. Training Results

**Figure 22:**
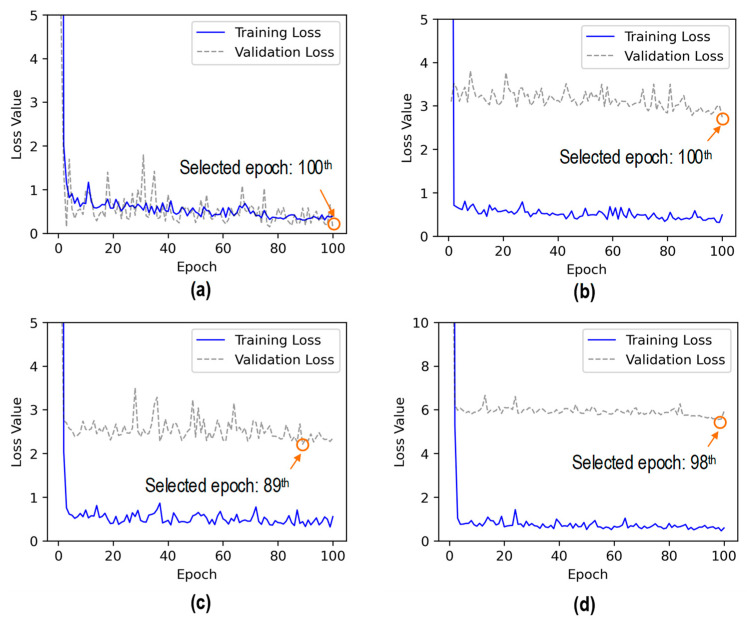
Loss values of 2D CNN model for untrained cases: (**a**) Case 1 (untrained S_2_); (**b**) Case 2 (untrained S_2_, S_4_); (**c**) Case 3 (untrained S_2_, S_3_); (**d**) Case 4 (untrained S_1_, S_3_, S_5_).

Figure 22 shows the loss values of the 2D CNN model during 100 epochs for Case 1 (untrained S_2_) and Case 2 (untrained S_2_ and S_4_). In Case 1, the training loss rapidly decreased in the initial 4 epochs and continued to decline with slight variations until the end. The validation loss rapidly dropped in the initial 4 epochs and fluctuated throughout the rest of the learning process. The training time was 30.2 s. The lowest validation loss was nearly 0.12 at the 100^th^ epoch. Thus, the trained 2D CNN model at the 100^th^ epoch was selected for further investigation.

In Case 2, the training loss dropped sharply in the initial 3 epochs and then progressively decreased with some variations until 100 epochs. The validation loss showed a quick decrease in the initial 2 epochs. Afterward, the validation loss experienced fluctuations and reduction until the 100^th^ epoch. The training time was 24.6 s. The lowest validation loss was nearly 2.78 at the 100^th^ epoch. Thus, the trained 2D CNN model at the 100^th^ epoch was selected to evaluate its accuracy on the testing set. 

In Case 3, the training loss dropped significantly in the initial 4 epochs and then fluctuated until the 100^th^ epoch. The validation loss showed a quick decrease in the initial 2 epochs. Afterward, the validation loss experienced fluctuations until the 100^th^ epoch. The training time was 25.1 s. The lowest validation loss was nearly 2.21 at the 89^th^ epoch. Thus, the trained 2D CNN model at the 89^th^ epoch was selected to evaluate its accuracy on the testing set.

In Case 4, the training loss dropped sharply in the initial 4 epochs and then slightly decreased with fluctuation until the 100^th^ epoch. The validation loss showed a quick decrease in the initial 2 epochs. Afterward, the validation loss experienced fluctuations until the 100^th^ epoch. The training time was 21.5 s. The lowest validation loss was nearly 5.56 at the 98^th^ epoch. Thus, the trained 2D CNN model at the 98^th^ epoch was selected to evaluate its accuracy on the testing set.

#### 4.2.3. Stress Estimation

**Figure 23:**
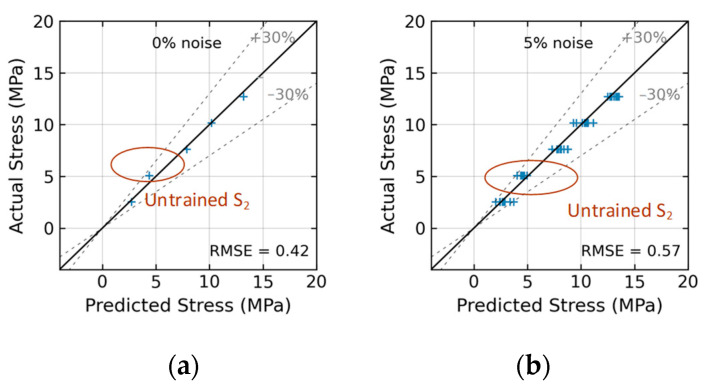
Stress estimation of 2D CNN model for Case 1 (untrained S_2_): (**a**) 0% noise; (**b**) 5% noise.

Figure 23 shows the stress estimation of the trained 2D CNN model for Case 1 (untrained S_2_). With low noise levels in the range of 0~5%, the model precisely estimated stress with an RMSE of about 0.42~0.57 (see Figure 23a,b). Although the EMI data at stress level S_2_ were excluded during the training process, the value of stress could still be predicted well by the 2D CNN model. The estimation error for stress level S_2_ was within 30% at the noise level of 5%. 

**Figure 24:**
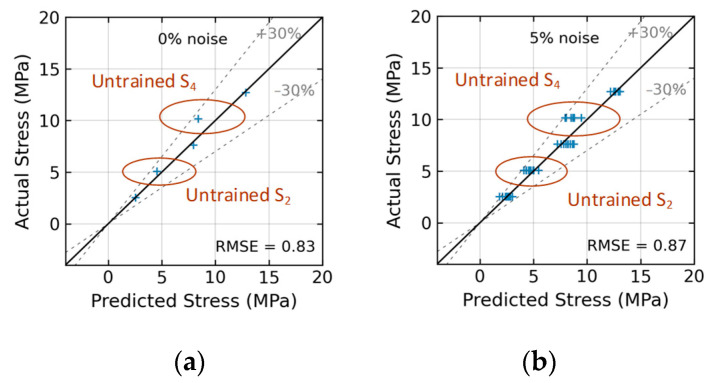
Stress estimation of 2D CNN model for Case 2 (untrained S_2_, S_4_): (**a**) 0% noise; (**b**) 5% noise.

Figure 24 shows the stress estimation of the trained 2D CNN model for Case 2 (untrained S_2_, S_4_). With low noise levels in the range of 0~5%, the model estimated stress levels with an RMSE of about 0.83~0.87. The accuracy of stress prediction on levels S_2_ and S_4_ decreased when these levels were excluded from the training process of the 2D CNN model. However, the prediction errors for these stress levels were still within 30% at the 5% noise level.

**Figure 25:**
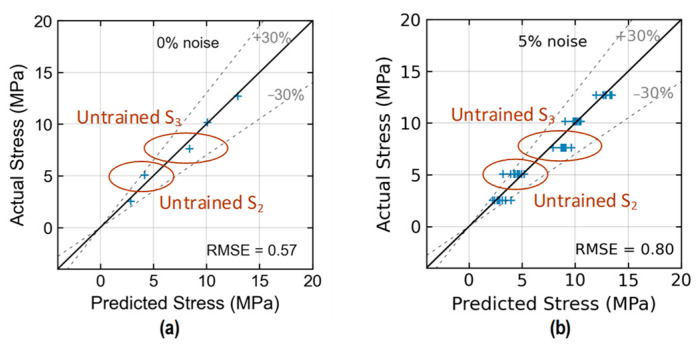
Stress estimation of 2D CNN model for Case 3 (untrained S_2_, S_3_): (**a**) 0% noise; (**b**) 5% noise.

Figure 25 shows the stress estimation of the trained 2D CNN model for Case 3 (untrained S_2_, S_3_). With low noise levels in the range of 0~5%, the model estimated stress levels with an RMSE of about 0.57~0.80. The accuracy of stress prediction on levels S_2_ and S_3_ decreased when these levels were excluded from the training process of the 2D CNN model. Some prediction errors of stress level S_2_ were over 30% at the 5% noise level. 

**Figure 26:**
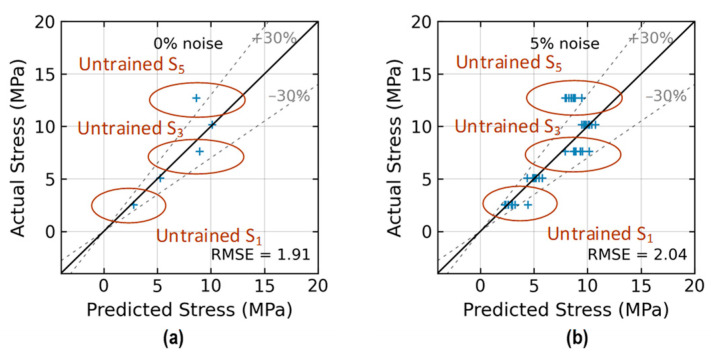
Stress estimation of 2D CNN model for Case 4 (untrained S_1_, S_3_, S_5_): (**a**) 0% noise; (**b**) 5% noise.

Figure 26 shows the stress estimation of the trained 2D CNN model for Case 4 (untrained S_1_, S_3_, S_5_). With low noise levels in the range of 0~5%, the model estimated stress levels with an RMSE of about 1.91~2.04. The accuracy of stress prediction on levels S_1_, S_3_, and S_5_ decreased when these levels were excluded from the training process of the 2D CNN model. Some prediction errors for stress levels S_1_ and S_5_ were over 30% at the 5% noise level.

**Figure 27:**
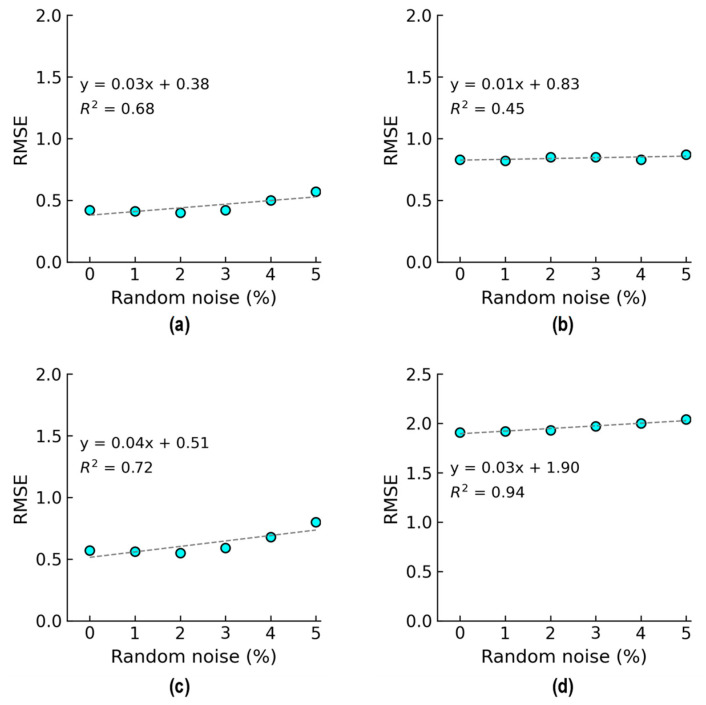
RMSE comparison of 2D CNN model for stress estimation: (**a**) Case 1 (untrained S_2_); (**b**) Case 2 (untrained S_2_, S_4_); (**c**) Case 3 (untrained S_2_, S_3_); (**d**) Case 4 (untrained S_1_, S_3_, S_5_).

Figure 27 shows the relationships between the RMSE and noise levels. For untrained S_2_ (see Figure 27a), the RMSE accounted for 0.41 at the 1% noise level and around 0.57 at the 5% noise level. For untrained S_2_ and S_4_ (see Figure 27b), the RMSE accounted for 0.82 at the 1% noise level and 0.87 at the 5% noise level. For untrained S_2_ and S_3_ (see Figure 27c), the RMSE accounted for 0.56 at the 1% noise level and 0.80 at the 5% noise level. For untrained S_1_, S_3_, and S_5_ (see Figure 27d), the RMSE accounted for 1.92 at the 1% noise level and 2.04 at the 5% noise level. Generally, a higher RMSE was attributed to a lack of training data. It was observed that the RMSEs in prediction in Cases 2, 3, and 4 were higher than that in Case 1. In Cases 2 and 4, the RMSEs in prediction increased insignificantly from 0 to 5% noise. Generally, as the number of data in the training sets decreased, the accuracy of stress estimation by the 2D CNN model declined.

#### 4.2.4. Damage Identification

##### Damage Identification Results

**Figure 28:**
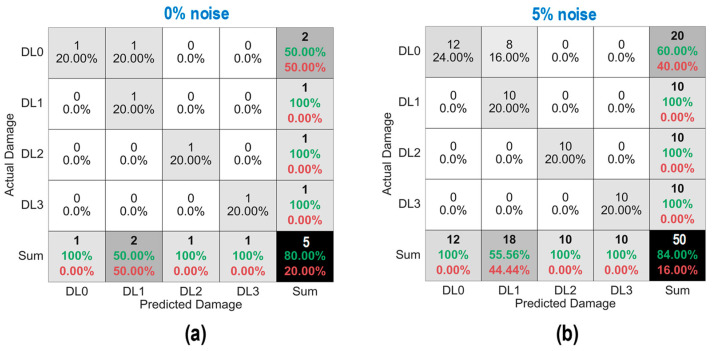
Damage identification of 2D CNN model for Case 1 (untrained S_2_): (**a**) 0% noise; (**b**) 5% noise.

As shown in Figure 28, the accuracy of damage identification by the trained 2D CNN model was assessed for Case 1 (untrained S_2_). With the 0% noise level (see Figure 28a), 100% of “DL1–DL3” was accurately classified, and only 50.0% of “DL0” was correctly classified. The precision for “DL1” was reduced to 50.0%. The precision for “DL0”, “DL2”, and “DL3” was 100%. The overall accuracy of the model reached 80.0%. 

With the 5% noise level (see Figure 28b), 100% of “DL1–DL3” was accurately classified, and 60% of “DL0” was correctly classified. The precision for “DL1” was about 55.5%, and the precision for “DL0”, “DL2”, and “DL3” was 100%. The overall accuracy of the model was 84.0%. The 2D CNN model incorrectly classified the damage status “DL0” as “DL1” at the 0% and 5% noise levels, indicating that the accuracy of the 2D CNN model could be affected significantly by untrained stress–damage EMI data.

**Figure 29:**
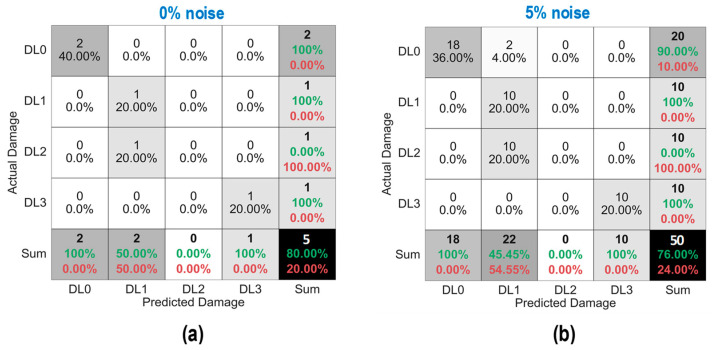
Damage identification of 2D CNN model for Case 2 (untrained S_2_, S_4_): (**a**) 0% noise; (**b**) 5% noise.

As shown in Figure 29, the accuracy of damage identification by the trained 2D CNN model was assessed for Case 2 (untrained S_2_, S_4_). With the 0% noise level (see Figure 29a), the model accurately classified “DL0”, “DL1”, and “DL3”, while it failed to classify “DL2”. The precision for “DL1” and “DL2” was 50.0% and 0.0%, respectively. The precision for “DL0” and “DL3” was 100%. The overall accuracy of the model reached 80.0%. 

With the 5% noise level (see Figure 29b), 100% of “DL1” and “DL3” was correctly detected, 90.0% of “DL0” was correctly detected, and 100% of “DL2” was incorrectly classified as “DL1”. The precision for “DL1” and “DL2” was about 45.4% and 0.0%, respectively, and that of “DL0” and “DL3” accounted for 100%. The overall accuracy of the model reached 76.0%. The 2D CNN model failed to detect “DL2” at the 0% and 5% noise levels. This could be induced by the lack of training data at damage level “DL2”.

**Figure 30:**
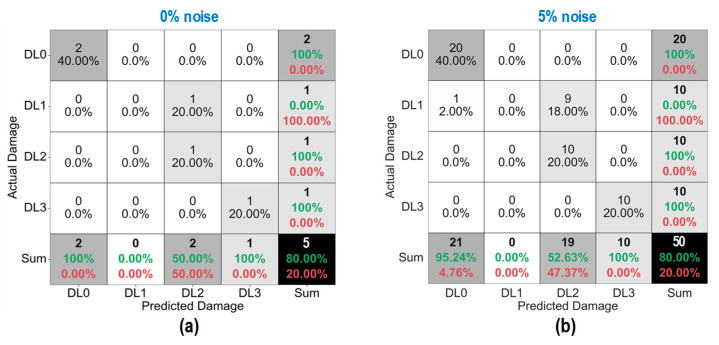
Damage identification of 2D CNN model for Case 3 (untrained S_2_, S_3_): (**a**) 0% noise; (**b**) 5% noise.

As shown in Figure 30, the accuracy of damage identification by the trained 2D CNN model was assessed for Case 3 (untrained S_2_, S_3_). With the 0% noise level (see Figure 30a), the model accurately classified “DL0”, “DL2”, and “DL3”, while it failed to classify “DL1”. The precision for “DL1” and “DL2” was 0.0% and 50.0%, respectively. The precision for “DL0” and “DL3” was 100%. The overall accuracy of the model reached 80.0%. With the 5% noise level (see Figure 30b), 100% of “DL0”, “DL2”, and “DL3” was correctly detected. Additionally, 100% of “DL1” was incorrectly classified as “DL0” and “DL2”. The precision for “DL0”, “DL1”, and “DL2” was about 95.24%, 0.0%, and 52.63%, respectively, and that of “DL3” accounted for 100%. The overall accuracy of the model reached 80.0%. The 2D CNN model failed to detect “DL1” at the 0% and 5% noise levels. This could be induced by the lack of training data at damage level “DL1”.

**Figure 31:**
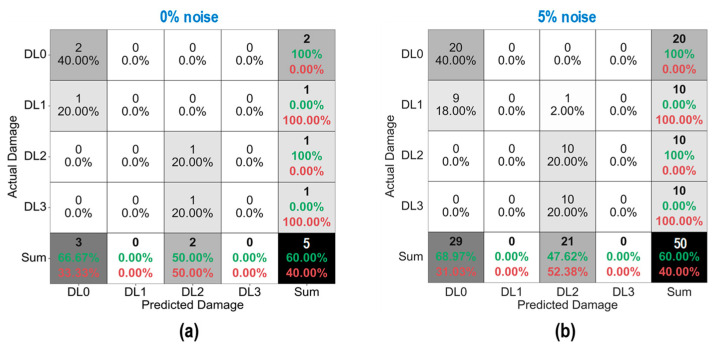
Damage identification of 2D CNN model for Case 4 (untrained S_1_, S_3_, S_5_): (**a**) 0% noise; (**b**) 5% noise.

As shown in Figure 31, the accuracy of damage identification by the trained 2D CNN model was assessed for Case 4 (untrained S_1_, S_3_, S_5_). With the 0% noise level (see Figure 31a), the model accurately classified “DL0” and “DL2”, while it failed to classify “DL1” and “DL3”. The precision for “DL0” and “DL2” was 66.67% and 50.0%, respectively. The precision for “DL1” and “DL3” was 0.0%. The overall accuracy of the model reached 60.0%. With the 5% noise level (see Figure 31b), 100% of “DL0” and “DL2” was correctly detected. Additionally, 100% of “DL1” was incorrectly classified as “DL0” and “DL2”, and 100% of “DL3” was incorrectly classified as “DL2”. The precision for “DL0” and “DL2” was about 68.97% and 47.62%, respectively, and that of “DL1” and “DL3” accounted for 0.0%. The overall accuracy of the model reached 60.0%. The 2D CNN model failed to detect “DL1” and “DL3” at the 0% and 5% noise levels. This could be induced by the lack of training data at damage levels “DL1” and “DL3”.

**Figure 32:**
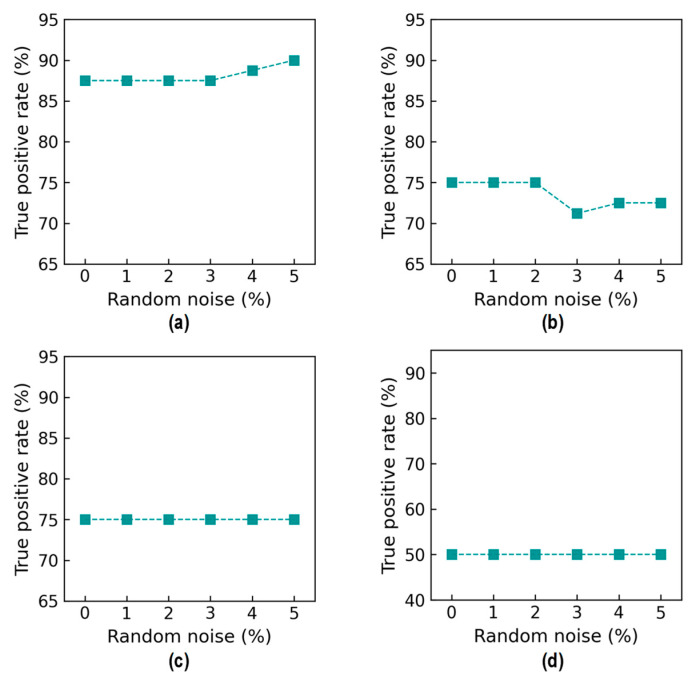
Accuracy of damage identification by 2D CNN model: true positive rate. (**a**) Case 1 (untrained S_2_); (**b**) Case 2 (untrained S_2_, S_4_); (**c**) Case 3 (untrained S_2_, S_3_); (**d**) Case 4 (untrained S_1_, S_3_, S_5_).

Figure 32 shows true positive rate (TPR) values. For Case 1 (untrained S_2_), the TPR was 87.5% from the 0% to 2% noise levels, and it slightly increased by 2.0% at 5% noise (see Figure 32a). For Case 2 (untrained S_2_ and S_4_), the TPR reached 75% upon the 0% to 2% noise level increase (see Figure 32b). The TPR obtained the lowest value (with 71% at 3% noise) before achieving 72.0% at 5% noise. The reduction in the TPR came from the false identification of “DL0” as “DL1” (see Figure 28). For Case 3 (untrained S_2_ and S_3_), the TPR reached 75.0% upon the 0% to 5% noise level increase (see Figure 32c). For Case 4 (untrained S_1_, S_3_, and S_5_), the TPR reached 50.0% upon the 0% to 5% noise level increase (see Figure 32d). 

**Figure 33:**
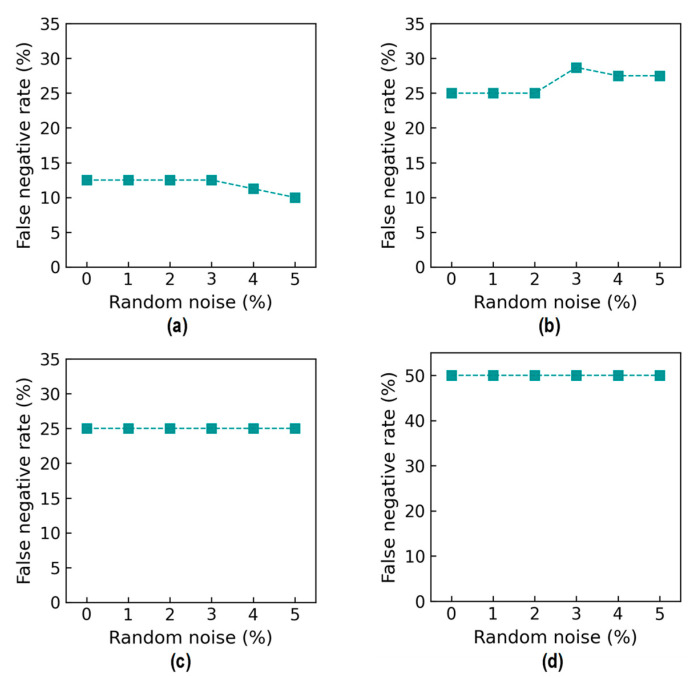
Accuracy of damage identification by 2D CNN model: false negative rate. (**a**) Case 1 (untrained S_2_); (**b**) Case 2 (untrained S_2_, S_4_); (**c**) Case 3 (untrained S_2_, S_3_); (**d**) Case 4 (untrained S_1_, S_3_, S_5_).

Figure 33 shows false negative rate (FNR) values. For Case 1 (untrained S_2_), the FNR was 12.5% from the 0% to 2% noise levels, and it slightly decreased to 2% at 5% noise (see Figure 33a). For Case 2 (untrained S_2_ and S_4_), the FNR was 25% from the 0% to 2% noise levels, followed by a small increase to about 28.1% at the 5% noise level (see Figure 33b). For Case 3 (untrained S_2_ and S_3_), the FNR was 25% from the 0% to 5% noise levels (see Figure 33c). For Case 4 (untrained S_1_, S_3_, and S_5_), the FNR was 50% from the 0% to 5% noise levels (see Figure 33d). 

**Figure 34:**
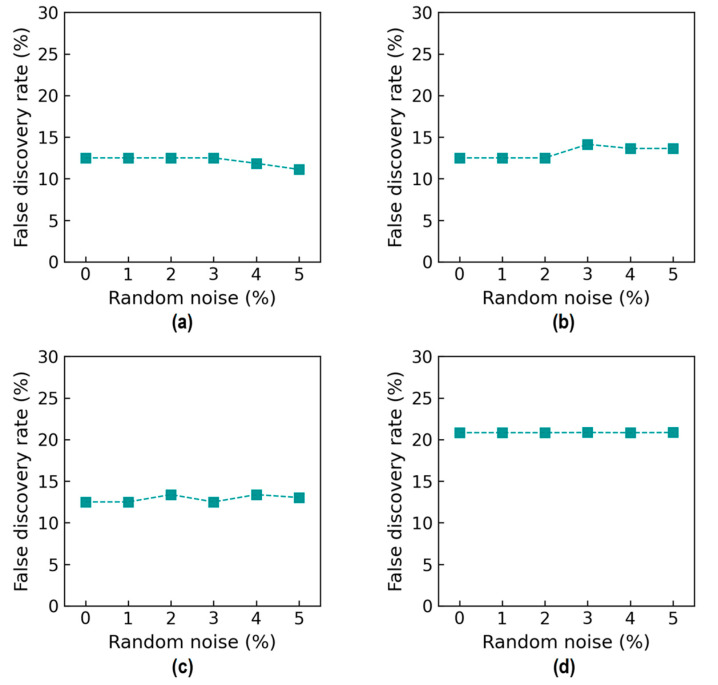
Accuracy of damage identification by 2D CNN model: false discovery rate. (**a**) Case 1 (untrained S_2_); (**b**) Case 2 (untrained S_2_, S_4_); (**c**) Case 3 (untrained S_2_, S_3_); (**d**) Case 4 (untrained S_1_, S_3_, S_5_).

Figure 34 shows the accuracy of damage identification in terms of the false discovery rate (FDR). For Case 1 (untrained S_2_), the FDR was 12.5% from the 0% to 2% noise levels (see Figure 34a), and it slightly decreased to about 11.1% at 5% noise. For Case 2 (untrained S_2_ and S_4_), the FDR was 12.5% from the 0% to 2% noise levels, followed by a small increase to about 13.6% at 5% noise (see Figure 34b). For Case 3 (untrained S_2_ and S_3_), the FDR fluctuated around 12.5% from the 0% to 5% noise levels (see Figure 34c). For Case 4 (untrained S_1_, S_3_, and S_5_), the FDR was around 20.8% from the 0% to 5% noise levels (see Figure 34d). It was observed that the 2D CNN model retained good precision (larger than 75.0%) even after excluding some stress levels from the training dataset.

##### Discussion on Damage Identification Results

**Figure 35:**
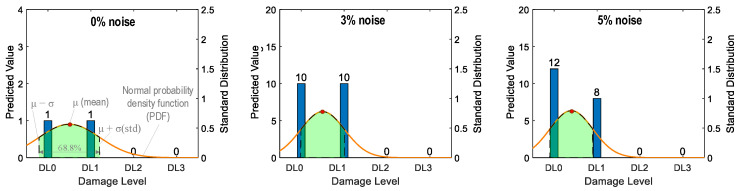
Probability assessment of damage identification results: Case 1 (untrained S_2_).

Figure 35 shows the probability assessment of the damage identification results for Case 1 (untrained S_2_). The stress level S_2_ corresponding to a part of damage level “DL0” was excluded from the training dataset. The x-axis shows four categories of damage levels “DL0”–“DL3”. The y-axes on the left and on the right indicate the predicted value and standard distribution, respectively. The normal probability density function (PDF) [42] is plotted on an orange line. 

The shaded area indicates the interval that lies within one standard deviation (σ) from the mean (μ). It encompassed 68.8% of the observed values from the central tendency of the prediction. For the case of partially untrained “DL0”, the green-shaded region indicates damage levels “DL0” and “DL1” at 0% noise (see Figure 35). At 3% noise, the green-shaded region also indicates that damage levels fell between “DL0” and “DL1”. However, at 5% noise, the shaded region with the mean value tended towards “DL0”.

**Figure 36:**
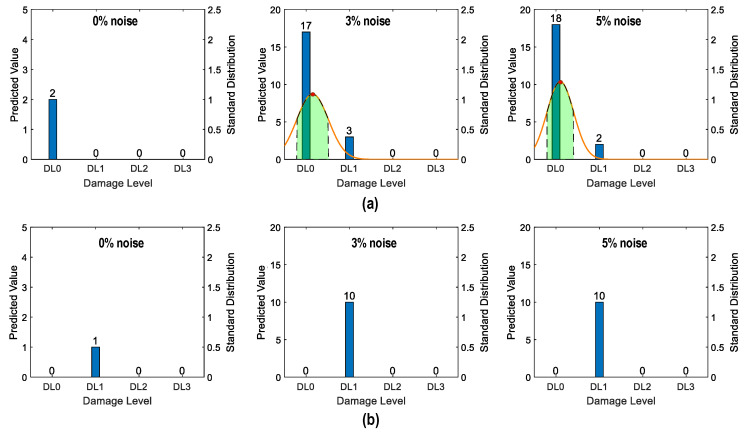
Probability assessment of damage identification results: Case 2 (untrained S_2_, S_4_). (**a**) Untrained “DL0” (partially); (**b**) untrained “DL2”.

Figure 36 shows the probability assessment of the damage identification results for Case 2 (untrained S_2_, S_4_). The stress levels S_2_ (a part of damage level “DL0”) and S_4_ (damage level “DL2”) were excluded from the training dataset. Figure 36a shows the probability assessment for partially untrained “DL0”. At 0% noise, all prediction results indicated that the damage level belonged to “DL0”. Their normal PDF chart was not displayed. With 3% and 5% noise, the green-shaded region indicates that the damage level belonged to “DL0”.

Figure 36b shows the probability assessment for untrained “DL2”. With 0%, 3%, and 5% noise, all prediction results indicated that the damage level belonged to “DL1”. The model mistakenly identified “DL2” as “DL1” because the untrained EMI features from damage level “DL2” had a high similarity to those from damage level “DL1”.

**Figure 37:**
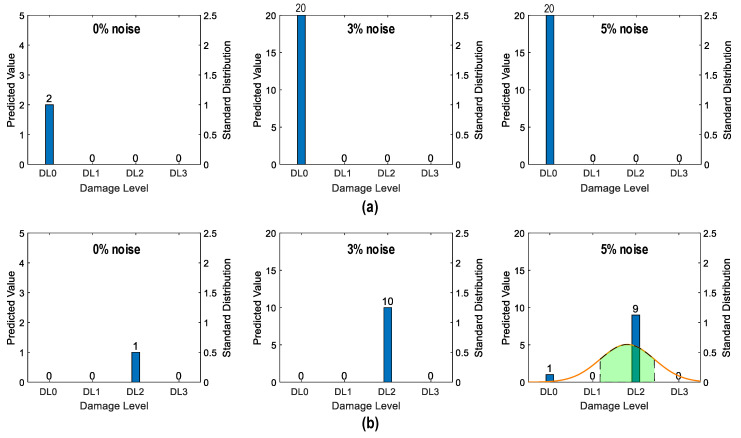
Probability assessment of damage identification results: Case 3 (untrained S_2_, S_3_). (**a**) Untrained “DL0” (partially); (**b**) untrained “DL1”.

Figure 37 shows the probability assessment of the damage identification results for Case 3 (untrained S_2_, S_3_). The stress levels S_2_ (a part of damage level “DL0”) and S_3_ (damage level “DL1”) were excluded from the training dataset. Figure 37a shows the probability assessment for partially untrained “DL0”. With 0%, 3%, and 5% noise, all prediction results indicated that the damage level belonged to “DL0”. Figure 37b shows the probability assessment for untrained “DL1”. With 0% and 3% noise, all prediction results indicated that the damage level belonged to “DL2”. With 5% noise, the green-shaded region indicated that the damage level belonged to “DL2”. The model mistakenly identified “DL2” as “DL1” because the untrained EMI features from damage level “DL1” had a high similarity to those from damage level “DL2”.

**Figure 38:**
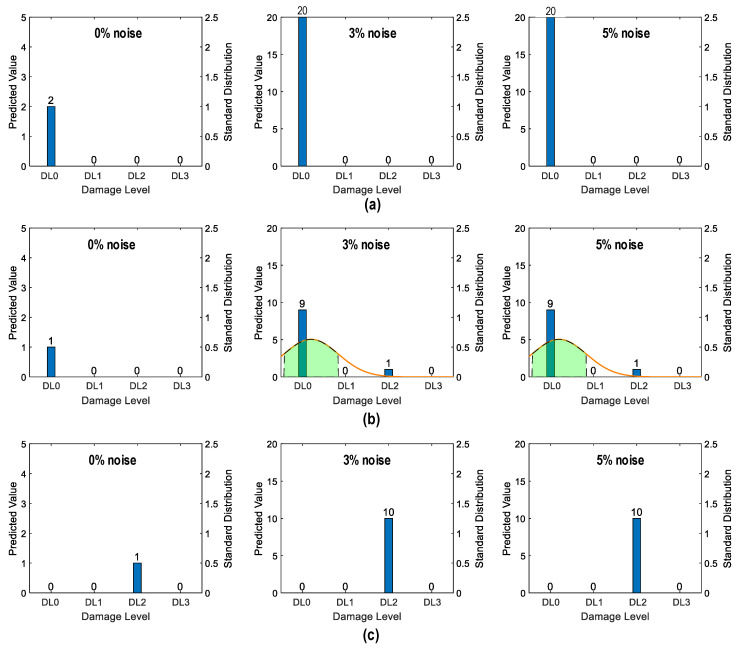
Probability assessment of damage identification results: Case 4 (untrained S_1_, S_3_, S_5_). (**a**) Untrained “DL0” (partially); (**b**) untrained “DL1”; (**c**) untrained “DL3”.

Figure 38 shows the probability assessment of the damage identification results for Case 4 (untrained S_1_, S_3_, S_5_). The stress levels S_1_ (a part of damage level “DL0”), S_3_ (damage level “DL1”), and S_5_ (damage level “DL3”) were excluded from the training dataset. Figure 38a shows the probability assessment for partially untrained “DL0”. With 0%, 3%, and 5% noise, all prediction results indicated that the damage level belonged to “DL0”. Figure 38b shows the probability assessment for untrained “DL1”. With 0% noise, all prediction results indicated that the damage level belonged to “DL0”. The normal PDF chart was not displayed. With 3% and 5% noise, the green-shaded region indicated that the damage level belonged to “DL0”. Figure 38c shows the probability assessment for untrained “DL3”. With 0%, 3%, and 5% noise, all prediction results indicated that the damage level belonged to “DL2”.

The visualization emphasized the model’s performance with partially untrained data, indicating its proficiency in identifying untrained damage levels to the closest trained levels.

## 5. Conclusions

This study developed a concrete stress and damage estimation method via EMI responses by a capsule-like smart aggregate (CSA) embedded in a concrete cylinder and a 2D CNN-based deep classification and regression learning model. The EMI damage measurement model was presented for the CSA-embedded concrete body under compressive load. The 2D CNN model was developed to simultaneously estimate concrete stress and damage in the concrete cylinder. The feasibility of the developed model was further investigated under the effect of noises and the untrained stress–damage EMI data. 

The following conclusions can be found from the analyzed results. Firstly, the analysis of EMI signals from the CSA-embedded concrete cylinder under z-direction compression revealed the non-linear behavior corresponding to various concrete damage levels. These non-linearities caused variations in the identified concrete damage status, potentially affecting the effectiveness of statistical damage diagnosis metrics. Secondly, the proposed model successfully extracted the stress and damage-sensitive features from the EMI data by the CSA sensor. The model accurately estimated the concrete stress levels (in MPa unit) and identified the concrete damage levels with small errors. Thirdly, the accuracy of stress estimation and damage identification by the proposed model was noticeably affected by the addition of noise to the CSA’s stress–damage EMI signals. In addition, the accuracy decreased when the stress–damage EMI data were excluded more from the training sets.

Despite the promising results, future studies are still needed for the following: (1) The method’s adaptability to various stress and damage types, concrete mixtures, and structural forms will be explored further. Transfer learning techniques will be applied to retrain the 2D CNN model for new applications efficiently. Since the model was already well trained on compressive stress–damage EMI data, it can be easily retrained with limited data for other stress and damage scenarios and concrete structures. (2) The architecture and hyper-parameters of the proposed model should be optimized to estimate concrete stress and damage more effectively. (3) Hybrid deep learning models should be considered to improve the accuracy of stress and damage monitoring. (4) The application of the proposed 2D CNN model for damage prediction in full-scale CSA-embedded concrete structures should be implemented. (5) Comparative studies with other state-of-the-art deep learning techniques [43] (i.e., RNN, LSTM, GRU, etc.) should be conducted to demonstrate the superior performance of the current method. (6) By redesigning the current architecture with advanced learning mechanisms [44] and data pre-processing methods [45], the proposed model can potentially be extended to a continual learning problem to adapt the dynamic data distributions of the CSA’s EMI responses over time. With continual learning application, the adaptive model could address complex problems in sensor fault diagnosis [46] or the influence of temperature variations on EMI responses [47], where EMI data exhibit non-linear changes over time.

## Figures and Tables

**Figure A1:**
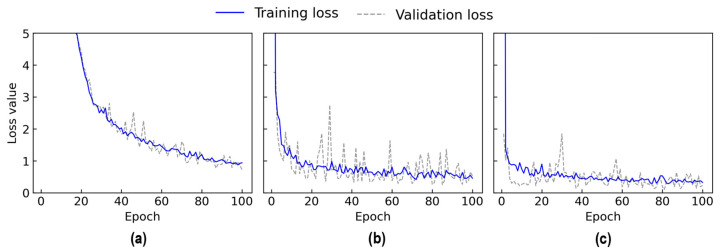
Training and validation loss of three 2D CNN architectures: (**a**) M1; (**b**) M2; (**c**) M3.

**Table 1:** Information on 2D CNN layers.

No	Type	Depth	Filter	Stride	No	Type	Depth	Filter	Stride
1	Conv1	18	3 × 3	1	8	Maxpool2	-	2 × 2	2
2	ReLU	-	-	-	9	GAP	-	-	-
3	Conv2	18	3 × 3	1	10	Fc_1_	1	-	-
4	ReLU	-		-	11	Fc_2_	4	-	-
5	Maxpool1	-	2 × 2	2	12	Regression	-	-	-
6	Conv3	18	3 × 3	1	13	Classification	-	-	-
7	ReLU	-	-	-					

**Table 2:** Material properties for x-CSA sensor-embedded concrete cylinder.

Properties	Aluminum (6061-T6)	PZT 5A	Epoxy Layer	Concrete
Mass density, *ρ* (kg/m^3^)	2700	7750	1090	2400
Young’s modulus, *E* (GPa)	68.9	62.1	0.75	25.43
Poisson’s ratio, *ν*	0.33	0.35	0.3	0.2
Dielectric loss factor, *δ*	0.02	0.015	0.02	
Yield strength, *σ_y_* (MPa)	241			
Compressive strength, *σ_c_* (MPa)			32.3	25.3
Damping loss factor, *η*		0.0125		
Dielectric constant, ε_33_^T^ (F/m)		1.53 × 10^−8^		
Coupling constant, *d*_31_ (m/V)		−1.71 × 10^−10^		

**Table 3:** Assigned labels of stress–damage EMI data for 2D CNN-based regression and classification model.

Stress Level	Observed Concrete Damage	Assigned Label	
		Stress (MPa)	Damage Level
S_1_	No damage	2.53	DL0
S_2_	No damage	5.07	DL0
S_3_	Crack initiation	7.61	DL1
S_4_	Crack propagation and spalling	10.15	DL2
S_5_	Failure	12.68	DL3

**Table 4:** Data cases for evaluating 2D CNN model.

Case	Scenario	Assigned Label		
		Training Set	Validation Set	Testing Set
1	Untrained S_2_	192	4	255
2	Untrained S_2_, S_4_	144	3	
3	Untrained S_2_, S_3_	144	3	
4	Untrained S_1_, S_3_, S_5_	96	2	

## Data Availability

Data are available on reasonable request from the corresponding author.

## Appendix A. Comparison of 2D CNN Architectures

A preliminary study was conducted to select an appropriate 2D CNN architecture for concrete stress and damage monitoring. Three 2D CNN architectures (M1–M3) were designed based on a previous well-established 2D CNN model [37]. Then, the training performance of the three architectures was compared using the training data in Section 4.1.

The specifications of M1–M3 are depicted in Table A1. While the input and output of these architectures are identical, their depth is different. Architecture M1 was constructed with a convolution layer (Conv1), as presented in Table A1. The ReLU, Maxpool1, and GAP layers orderly follow the Conv1 layer. Two Fc layers were configured parallel to the GAP layer. Architecture M2 was constructed by adding a convolution layer (Conv2) and a ReLU layer. The layers could increase the non-linear transformation of the input data and enhance the convergence speed in the training procedure [39]. Architecture M3 was built by increasing the depth of M2. Specifically, a set of three sequential layers of M1, which were layer 3 (Conv), layer 4 (ReLU), and layer 5 (Maxpool), was doubled to create M3. Further details regarding the depth, filters, and strides of each layer in M1–M3 are described in Table A1.

Figure A1 shows the training and validation loss value of the three architectures during the learning procedure of 100 epochs. As seen in the figure, the loss values of training and validation dropped in the 100 epochs and converged until the last epoch. Among the three architectures, M3 had the lowest loss during the learning process, followed by M2 and M1. The M3 architecture was effectively trained in 100 epochs, surpassing the other architectures in effectively extracting optimal damage features from EMI datasets. Therefore, the M3 architecture was selected for the 2D CNN-based concrete stress and damage prediction model.

**Table A1:** Information of three 2D CNN architectures (M1–M3).

No	Type	Depth	Filter	Stride	No	Type	Depth	Filter	Stride
Model M1									
1	Conv1	18	3 × 3	1	5	Fc_1_	1	-	-
2	ReLU	-	-	-	6	Fc_2_	4	-	-
3	Maxpool1	18	3 × 3	1	7	Regression	-	-	-
4	GAP	-	-	-	8	Classification	-	-	-
Model M2									
1	Conv1	18	3 × 3	1	6	GAP	-	-	-
2	ReLU	-	-	-	7	Fc_1_	1	-	-
3	Conv2	18	3 × 3	1	8	Fc_2_	4	-	-
4	ReLU	-	-	-	9	Regression	-	-	-
5	Maxpool1	-	2 × 2	2	10	Classification	-	-	-
Model M3 (see Table 1)									

## References

[1] Khan M.K.I., Lee C.K., Zhang Y.X., Rana M.M. (2020). Compressive behaviour of ECC confined concrete partially encased steel composite columns using high strength steel. Constr. Build. Mater..

[2] Smolana A., Klemczak B., Azenha M., Schlicke D. (2021). Experiences and analysis of the construction process of mass foundation slabs aimed at reducing the risk of early age cracks. J. Build. Eng..

[3] Suzuki T., Shiotani T., Ohtsu M. (2017). Evaluation of cracking damage in freeze-thawed concrete using acoustic emission and X-ray CT image. Constr. Build. Mater..

[4] Tan X., Abu-Obeidah A., Bao Y., Nassif H., Nasreddine W. (2021). Measurement and visualization of strains and cracks in CFRP post-tensioned fiber reinforced concrete beams using distributed fiber optic sensors. Autom. Constr..

[5] Ong C.-W., Yang Y., Naidu A.S.K., Lu Y., Soh C.K. (2002). Application of the electromechanical impedance method for the identification of in-situ stress in structures. Smart Struct. Devices Syst..

[6] Neild S.A., Williams M.S., Mcfadden P.D. (2005). Development of a Vibrating Wire Strain Gauge for Measuring Small Strains in Concrete Beams. Strain.

[7] Lee S.-J., Ahn D., You I., Yoo D.-Y., Kang Y.-S. (2020). Wireless cement-based sensor for self-monitoring of railway concrete infrastructures. Autom. Constr..

[8] Zhao X., Wen F., Chan T.-M., Cao S. (2019). Theoretical Stress–Strain Model for Concrete in Steel-Reinforced Concrete Columns. J. Struct. Eng..

[9] Huynh T.-C., Dang N.-L., Kim J.-T. (2017). Advances and Challenges in impedance-based structural health monitoring. Struct. Monit. Maint..

[10] Liang C., Sun F.P., Rogers C.A. (1994). Coupled Electro-Mechanical Analysis of Adaptive Material Systems-Determination of the Actuator Power Consumption and System Energy Transfer. J. Intell. Mater. Syst. Struct..

[11] Narayanan A., Kocherla A., Subramaniam K.V.L. (2018). PZT sensor array for local and distributed measurements of localized cracking in concrete. Smart Mater. Struct..

[12] Song G., Gu H., Mo Y.-L. (2008). Smart aggregates: Multi-functional sensors for concrete structures—A tutorial and a review. Smart Mater. Struct..

[13] Kong Q., Fan S., Mo Y.L., Song G. (2017). A novel embeddable spherical smart aggregate for structural health monitoring: Part II. Numerical and experimental verifications. Smart Mater. Struct..

[14] Li G., Luo M., Huang J., Li W. (2023). Early-age concrete strength monitoring using smart aggregate based on electromechanical impedance and machine learning. Mech. Syst. Signal Process..

[15] Pham Q.-Q., Dang N.-L., Ta Q.-B., Kim J.-T. (2021). Optimal Localization of Smart Aggregate Sensor for Concrete Damage Monitoring in PSC Anchorage Zone. Sensors.

[16] Lan C., Liu H., Zhuang S., Wang J., Li W., Lin G. (2024). Monitoring of crack repair in concrete using spherical smart aggregates based on electromechanical impedance (EMI) technique. Smart Mater. Struct..

[17] Pham Q.-Q., Ta Q.-B., Kim J.-T. (2022). Capsule-like Smart Aggregate with Pre-Determined Frequency Range for Impedance-Based Stress Monitoring. Sensors.

[18] Lim Y.Y., Smith S.T., Padilla R.V., Soh C.K. (2021). Monitoring of concrete curing using the electromechanical impedance technique: Review and path forward. Struct. Health Monit..

[19] Nguyen T.-T., Tuong Vy Phan T., Ho D.-D., Man Singh Pradhan A., Huynh T.-C. (2022). Deep learning-based autonomous damage-sensitive feature extraction for impedance-based prestress monitoring. Eng. Struct..

[20] Nguyen T.-T., Ta Q.-B., Ho D.-D., Kim J.-T., Huynh T.-C. (2023). A method for automated bolt-loosening monitoring and assessment using impedance technique and deep learning. Dev. Built Environ..

[21] Nguyen T.-T., Ho D.-D., Huynh T.-C. (2022). Electromechanical impedance-based prestress force prediction method using resonant frequency shifts and finite element modelling. Dev. Built Environ..

[22] Lee J., Kim H.S., Kim N., Ryu E.M., Kang J.W. (2019). Learning to Detect Cracks on Damaged Concrete Surfaces Using Two-Branched Convolutional Neural Network. Sensors.

[23] Ta Q.-B., Pham Q.-Q., Pham N.-L., Huynh T.-C., Kim J.-T. (2024). Smart Aggregate-Based Concrete Stress Monitoring via 1D CNN Deep Learning of Raw Impedance Signals. Struct. Control Health Monit..

[24] Ta Q.-B., Pham Q.-Q., Pham N.-L., Kim J.-T. (2024). Integrating the Capsule-like Smart Aggregate-Based EMI Technique with Deep Learning for Stress Assessment in Concrete. Sensors.

[25] Nguyen T.-T., Kim J.T., Ta Q.B., Ho D.D., Phan T.T.V., Huynh T.C. (2021). Deep learning-based functional assessment of piezoelectric-based smart interface under various degradations. Smart Struct. Syst..

[26] Yan Q., Liao X., Zhang C., Zhang Y., Luo S., Zhang D. (2022). Intelligent monitoring and assessment on early-age hydration and setting of cement mortar through an EMI-integrated neural network. Measurement.

[27] Ai D., Mo F., Han Y., Wen J. (2022). Automated identification of compressive stress and damage in concrete specimen using convolutional neural network learned electromechanical admittance. Eng. Struct..

[28] Ai D., Cheng J. (2023). A deep learning approach for electromechanical impedance based concrete structural damage quantification using two-dimensional convolutional neural network. Mech. Syst. Signal Process..

[29] Ai D., Mo F., Cheng J., Du L. (2023). Deep learning of electromechanical impedance for concrete structural damage identification using 1-D convolutional neural networks. Constr. Build. Mater..

[30] Nawy E.G. (1996). Prestressed Concrete. A Fundamental Approach.

[31] Baptista F.G., Filho J.V. (2010). Optimal Frequency Range Selection for PZT Transducers in Impedance-Based SHM Systems. IEEE Sens. J..

[32] Pham Q.-Q., Ta Q.-B., Park J.-H., Kim J.-T. (2022). Raspberry Pi Platform Wireless Sensor Node for Low-Frequency Impedance Responses of PZT Interface. Sensors.

[33] Kocherla A., Subramaniam K.V.L. (2020). Embedded smart PZT-based sensor for internal damage detection in concrete under applied compression. Measurement.

[34] Jalloh A. (2005). Effects of Piezoelectric (PZT) Sensor Bonding and the Characteristics of the Host Structure on Impedance Based Structural Health Monitoring.

[35] Min J., Park S., Yun C.-B. (2010). Impedance-based structural health monitoring using neural networks for autonomous frequency range selection. Smart Mater. Struct..

[36] Salman S., Liu X. (2019). Overfitting mechanism and avoidance in deep neural networks. arXiv.

[37] Lecun Y., Bottou L., Bengio Y., Haffner P. (1998). Gradient-based learning applied to document recognition. Proc. IEEE.

[38] Zhang Y., Wallace B. (2015). A sensitivity analysis of (and practitioners’ guide to) convolutional neural networks for sentence classification. arXiv.

[39] Lin M., Chen Q., Yan S. (2013). Network in network. arXiv.

[40] Goodfellow I., Bengio Y., Courville A. (2016). Deep Learning.

[41] Huynh T.C., Kim J.T. (2014). Impedance-Based Cable Force Monitoring in Tendon-Anchorage Using Portable PZT-Interface Technique. Math. Probl. Eng..

[42] Fisher L. (1990). Probability and statistics. Handbook of Applied Mathematics: Selected Results and Methods.

[43] Azimi M., Eslamlou A.D., Pekcan G. (2020). Data-Driven Structural Health Monitoring and Damage Detection through Deep Learning: State-of-the-Art Review. Sensors.

[44] Liu Z., Mao H., Wu C.-Y., Feichtenhofer C., Darrell T., Xie S. (2022). A ConvNet for the 2020s. arXiv.

[45] Ren X., Qin Y., Li B., Wang B., Yi X., Jia L. (2024). A core space gradient projection-based continual learning framework for remaining useful life prediction of machinery under variable operating conditions. Reliab. Eng. Syst. Saf..

[46] Ho D.D., Huynh T.C. (2022). Nondestructive crack detection in metal structures using impedance responses and artificial neural networks. Struct. Monit. Maint..

[47] Huynh T.C., Dang N.L., Kim J.-T. (2018). PCA-based filtering of temperature effect on impedance monitoring in prestressed tendon anchorage. Smart Struct. Syst..

